# Network pharmacology analysis combined with experimental validation to explore the therapeutic mechanism of salidroside on intestine ischemia reperfusion

**DOI:** 10.1042/BSR20230539

**Published:** 2023-08-25

**Authors:** Feng Chen, Yi-hong Chai, Fa Zhang, Yong-qiang Liu, Yan Zhang, Ya-jing Shi, Jian-ming Zhang, Yu-fang Leng

**Affiliations:** 1 The First Clinical Medical College of Lanzhou University, Lanzhou, 730000, GanSu Province, China; 2 Department of Anesthesiology, First Hospital of Lanzhou University, Lanzhou, 730000, GanSu Province, China; 3 Department of Urology, Gansu Provincial Hospital, Lanzhou, Gansu, China

**Keywords:** inflammation, intestinal ischemia-reperfusion injury, network pharmacology, Salidroside

## Abstract

Ethnopharmacological relevance: Salidroside (SAL), a phenolic natural product present in Rhodiola rosea, are commonly used in the treatment of various ischemic-hypoxic diseases, including intestinal ischemia–reperfusion (IR) injury. However, their efficacy and potential mechanisms in the treatment of intestinal IR injury have not been investigated.

Objective: The objective of the present study is to investigate the pharmacological mechanism of action of SAL on intestinal IR injury using a network pharmacology approach combined with experimental validation.

Methods: In the present study, we used the Traditional Chinese Medicine Systematic Pharmacology (TCMSP) database and analysis platform and Comparative Toxicogenomics Database (CTD) to predict possible target genes of SAL, collected relevant target genes of intestinal IR injury from GeneCards and DisGenet websites, and collected summary data to screen common target genes. Then, the protein–protein interaction (PPI) target network was constructed and analyzed by STRING database and Cytoscape 3.8.2 with the above intersecting genes. Then, gene ontology (GO) and Kyoto Encyclopedia of Genes and Genomes (KEGG) pathway analyses were performed and the component-target-pathway network was constructed, followed by the use of molecular docking and molecular dynamic simulation to verify the possible binding conformation between SAL and candidate targets to further explore the potential targets of SAL in the treatment of intestinal IR injury. Finally, an *in vivo* model of mouse superior mesenteric artery ligation was established to assess the anti-intestinal IR injury effect of SAL by assessing histopathological changes in mouse small intestine by HE staining, detecting inflammatory factor expression by ELISA kit, and detecting the expression of key protein targets by Western blotting.

Results: A total of 166 SAL target genes and 1740 disease-related targets were retrieved, and 88 overlapping proteins were obtained as potential therapeutic targets. The pathway enrichment analysis revealed that the pharmacological effects of SAL on intestinal IR injury were anti-hypoxic, anti-inflammatory and metabolic pathway related, and the molecular docking and molecular dynamic simulation results showed that the core bioactive components had good binding affinity for TXNIP and AMPK, and the immunoblotting results indicated that the expression levels of TXNIP and AMPK in the small intestinal tissues of mice in the drug-treated group compared with the model group were significantly changed.

Conclusion: SAL may target AMPK and TXNIP domains to act as a therapeutic agent for intestinal IR. These findings comprehensively reveal the potential therapeutic targets for SAL against intestinal IR and provide theoretical basis for the clinical application of SAL in the treatment of intestinal IR.

## Introduction

Intestinal ischemia reperfusion (IR) injury is a complex pathological process with multifactorial involvement due to multiple etiologies, which not only causes local damage to the intestine, but also disruption of the intestinal mucosal barrier caused by tissue hypoxia, inflammation and cellular infiltration is the ‘trigger point’ for systemic organ damage [1]. The increased permeability of the intestinal mucosa can lead to the translocation of intestinal bacteria and endotoxins, massive oxygen radical production, inflammatory cell infiltration, and release of inflammatory mediators and cytokines, causing damage to various distant organs such as the lung, liver and brain, resulting in systemic inflammatory response syndrome (SIRS) and even multi-organ failure (MOF) and death [2,3]. The incidence of acute mesenteric ischemia has been reported to be 0.1%, with an overall mortality rate of 50% and a mortality rate of 71% within 30 days in ICU patients [4]. Owing to the complexity of the pathological process of intestinal IR injury, single-target drugs are hard to reach a satisfactory therapeutic effect. Until now, specific drugs and therapeutic approaches for intestinal IR injury remain to be elucidated. Thus, the exploitation of multi-targeted drugs from traditional medicine for the treatment of intestinal IR injury has emerged as a popular research direction.

Rhodiola rosea, also known as ‘rose root’, ‘golden root’ or ‘arctic root’, belongs to the plant family Rosaceae. It is a perennial yellow-flowered herb that grows naturally in dry, sandy soils, sea cliffs and crevices in the Arctic regions of Europe and Asia (mainly Siberia) and the eastern seaboard of North America. Rhodiola rosea is a phytomedicinal plant with an adaptogenic effect that enhances the body’s non-specific resilience to physical and mental stress and normalizes its function [5]. The main pharmacologically active components isolated from various Rhodiola species are rhodiol glycosides and their glycosides (tyrosol), rosavin or rosavidine, pyridine, rhodiola and rhodiola. According to the available studies, most of the studies have focused on the traditional uses, phytochemistry and pharmacology of Rhodiola rosea [6]. Salidroside (SAL) is a phenolic product isolated from Rhodiola rosea with molecular formula C_14_H_20_O_7_. SAL possesses various pharmacological effects including anti-hypoxia, antioxidant, anti-stress, anti-fatigue, anti-inflammatory, anti-cancer, anti-metabolic dysregulation, anti-immune stimulation and neuroprotection [7–9], which can prevent and treat different diseases, such as cancer, atherosclerosis, Alzheimer’s disease, Parkinson’s disease, pneumonia and cardiovascular diseases [10–14] With the intensive studies, it has been identified that SAL can exert good organ protective effects through multi-target and multi-pathway mechanisms such as inhibition of apoptosis, oxidative stress and inflammatory response [15,16]. At this time, the specific mechanism of action of SAL against IR injury is not yet clarified, and the potential protective function of SAL in intestinal IR injury has not been extensively explored.

**Figure 1:**
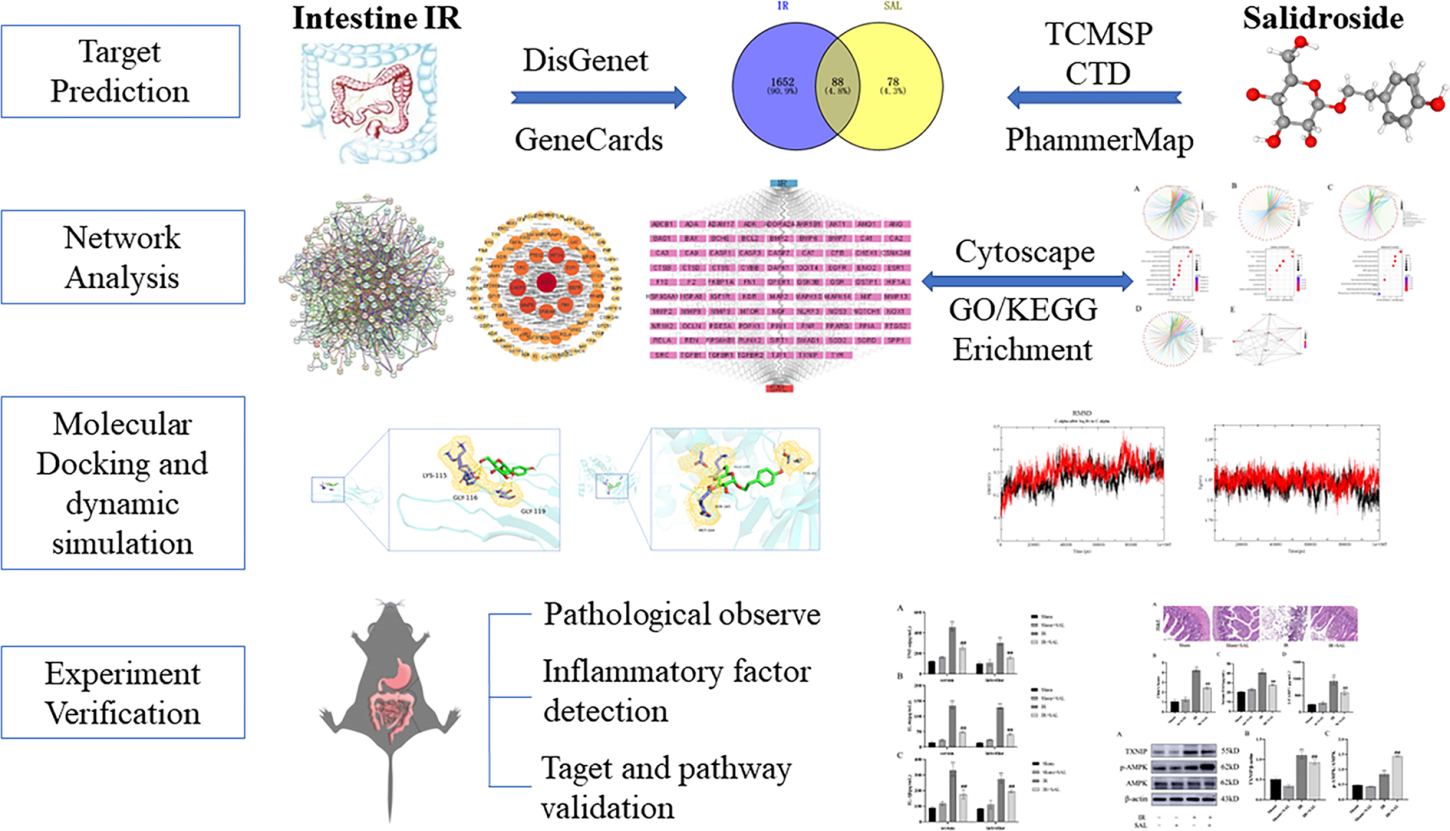
Flowchart of ideas for this study

Network pharmacology is a novel, promising and cost-effective approach to drug development based on bioinformatics, systems biology and polypharmacology, with integrity and systematization [17]. It examines the ‘compound-protein/gene-disease’ pathway by mapping drug targets and disease evidence-related molecules to biomolecular networks, analyzing the intricate relationships between biological systems, drug and diseases from a cyber perspective [18]. Network pharmacology has transformed the traditional concept of drug development from the current ‘one target, one drug’ research strategy to a ‘multi-target, multi-component’ approach, which is fully compatible with the integrity and systematization of Chinese herbal medicines and contributes to the clarification of the scientific basis of Chinese medicines. Network pharmacology has been broadly applied to uncover the potential mechanism of action of single components or herbal compound formulations [19]. Therefore, this work aims to utilize a network pharmacology approach to investigate the possible mechanisms by which SAL alleviates intestinal IR injury. Building upon the organ-protective effects of SAL, the ameliorative impact on intestinal IR has been demonstrated by establishing an *in vivo* model of intestinal IR injury in mice, along with the identification of critical protein targets using immunoblotting analysis. Our study offers a theoretical basis for the precise application of SAL and the development of multi-targeted drugs. The overall idea of the study is illustrated in Figure 1.

## Materials and methods

### Access to SAL and intestinal IR targets

By filtering the Traditional Chinese Medicine Systems Pharmacology database (TCMSP, https://old.tcmsp-e.com/index.php), Comparative Toxicogenomics Database (CTD, https://ctdbase.org/) and PhammerMap (http://lilab-ecust.cn/pharmmapper/submitfile.html) databases to generalize SAL-related target proteins. The collection of target genes related to intestinal IR injury was then performed by searching the DisGenet (https://www.disgenet.org/) and GeneCard (https://www.genecards.org/) databases to acquire disease-related genes. Due to the probable incomplete standardization of the nomenclature of targets obtained from different databases, the aforementioned targets were converted into standardized gene names using the Uniprot (https://www.genecards.org/) and restricted to the species ‘human’.

### Plotting a Venn Diagram

We used the online tool of Venny (2.0.1) to filter out SAL and intestinal IR intersection target genes by loading target genes of SAL and intestinal IR, automatically retrieving overlapping genes and drawing Venn diagrams.

### Construction of drug-disease target protein interaction network

The intersection targets were inserted into the String database (https://string-db.org/), the species was chosen as *Homo sapiens*, the minimization threshold was adjusted to high confidence 0.4, and the solitary targets with no linkage were excluded. The obtained protein interaction network data were inserted into Cytoscape 3.8.2 to create protein–protein interaction (PPI) network.

### Analysis of intersectional target pathways and functional enrichment

Gene Ontology (GO) and Kyoto Encyclopedia of Genes and Genomes (KEGG) pathway analyses were performed with DAVID database and visualized with R packages. These R packages were obtained by downloading from Bioconductor’s open source platform (https://www.bioconductor.org/). GO analysis was conducted as an in-depth exploration of biological processes (BP), molecular functions (MF) and cellular components (CC). A filter was set at *P*<0.05 for further analysis, and the top 20 terms with the highest concentration in GO and KEGG were selected and visualized.

### Construction of CTD network

We have built a CTD network by Cytoscape 3.8.2 using SAL, intestinal IR and their common targets as the study subjects. Nodes denote chemical substances (e.g. SAL), intersecting target genes and diseases (e.g. intestinal IR), while edges stand for interactions.

### Molecular docking

Molecular docking was carried out to validate potential target–drug interactions. The 2D structure of SAL (MOL003341) was downloaded from PubChem (https://pubchem.ncbi.nlm.nih.gov/), imported into ChemBio3D 19.0 software, and saved in mol2 format. The crystal structures of the core targets were downloaded from protein data bank (PDB https://www.rcsb.org/), including the thioredoxin-interacting protein (TXNIP) (PDB code: 4GEI) and the adenosine monophosphate-activated protein kinase (AMPK) (PDB code: 2H6D). The target proteins were imported into Pymol 2.5.0 to eliminate the proligands, water molecules, pre-treated such as hydrogenation and searching for active pockets using AutodockTools-1.5.7, and saved as pdbqt format. The selection of candidate proteins according to the degree values obtained from PPI was performed on Autodock vina 1.5.7 platform for docking. The localization points were defined using a grid of 40,40,40 with a spacing of 1.000 Å per grid and other parameters using default values. The lower the score and the higher the affinity. The proteins with the lowest scores were finally selected and visualized by Pymol 2.5.0.

### Molecular dynamic simulation

Molecular dynamics (MD) simulations [20] were performed using GROMACS software (2018.1) to validate the configuration of the molecular docking of the TXNIP–SAL complex. The missing hydrogen atoms in the initial structure of the ligand were appended under neutral conditions using Discovery Studio version 4.5 software. The topology of TXNIP was generated by employing GROMACS54a7 stand and the parameter files of small molecule SAL under GROMACS force field were available from ATB website (https://atb.uq.edu.au). The TXNIP system was placed in a cubic box and solventized using the SPC water model [21], while Na^+^/Cl^−^ was later included to make the system electrically neutral [22]. Prior to the MD simulation, the simulated system was optimized energetically using the steepest descent method in 50,000 steps, and the canonical ensemble (NVT) and constant-pressure (NPT) equilibria were performed before and after 100 ps, respectively, with a control temperature of 310 K and a pressure of 1.0 bar, while the long-range electrostatic force was treated by the PME (Particle Mesh Ewald) method. The MD simulation was finished under these conditions for 100 ns. Finally, the resulting data were plotted with the Xmgrace software.

### Experimental validation

#### Reagents and antibodies

Salidroside (MedChemExpress, Product No. 10338-51-9, purity >99.99%, molecular weight 300.30) is ordered commercially. Rabbit anti-p-AMPK (Thr 172) (1:1000, #2535, Cell Signaling Technology [CST], U.S.A.), rabbit anti-AMPK (1:1000, #5831, CST, U.S.A.) and TXNIP (1:1000, #14715, CST, U.S.A.).

#### Experimental models and pharmacotherapy

A total of 24 healthy male C57BL/6J mice, weighing 25 ± 2 g, were purchased from the Experimental Animal Center of Lanzhou University for this experiment. All animal experiments took place at Basic Laboratory of Lanzhou University. All experimental procedures were performed in accordance with the International Guide for the Care and Use of Laboratory Animals and were approved by the Animal Ethics Committee of the Lanzhou University. The animals were housed in well-ventilated SPF-rated rooms (temperature: 20 ± 2°C, humidity: 45–55%, 12-h light and dark cycle) and were fed food and water ad libitum. After 1 week of adaptive feeding, the mouse were randomly divided into four groups (*n*=6): sham-operated group (Sham group), SAL-treated group (SAL group), model group (IR group) and model + SAL-treated group (IR + SAL group). Mice in the IR and IR + SAL groups were constructed as mouse intestinal IR injury models, and mice in the Sham and SAL groups were only isolated from the superior mesenteric artery (SMA) without ligation. Mice in the SAL and IR + SAL groups were injected intraperitoneally with SAL (30 mg/kg) for 7 days before operation, and the mice in the Sham and IR groups were injected with equal amounts of saline at the same time point. After the mice in each group were executed, blood specimens and intestinal tissue specimens (10 cm from the ileocecal area) were gathered for examination.

The mice in model were intraperitoneally injected with pentobarbital (30 mg/kg) to anesthetize the mice, the mice were immobilized in the supine position on a small animal test bench. The abdominal region was prepared for surgery and disinfected by wiping with 75% alcohol. An approximately 2 cm long incision was created along the midline of the abdomen, and the intestine was exposed in layers, and the SMA was fully exposed and isolated using toothless forceps, dissection forceps, and a glass splitting needle, SMA was clamped with a saline-wetted non-invasive micro-arterial clip to block the blood flow. Visually, the color of intestinal tissue turns from bright red to pale white, signifying successful ischemia. Warm sterile gauze containing saline is used to cover the wound. The wound was covered with warm sterile gauze containing saline. After 45 min of ischemia, the non-invasive micro-artery clamps were removed to restore blood perfusion to the intestine. Visually a successful reperfusion is indicated by a change from pale to bright red intestinal tissue. The abdominal cavity of the mice was carefully examined to make sure there were no abnormalities, and then the abdomen was closed one by one with sterile silk thread. After 120 min of reperfusion, the mice were executed.

All procedures for this study were performed and agreed upon in accordance with internationally accepted principles for the use and care of laboratory animals, and the protocol was approved by the Animal Protection and Ethics Committee of the First Hospital of Lanzhou University.

#### Histological analysis

Intestinal tissues were captured, fixed in 4% paraformaldehyde, and embedded in paraffin blocks. The degree of intestinal histopathological lesion in mice was assessed by double-blind method with hematoxylin and eosin (H&E) staining, referring to Chiu’s score [23].

#### Enzyme-linked immunosorbent assay

Measurement of serum and intestinal tissue levels of interleukin (IL)-6, IL-1β and tumor necrosis factor (TNF)-α. ELISA Kit (Mlbio, Shanghai, China) was used according to the manufacturer’s instructions.

#### Western blotting

The bowel tissue was extracted from −80°C and immediately placed on ice. The tissue was sliced into small pieces, placed in Eppendorf tubes, and lysed on ice for 30 min with 100 μL/10kg lysis buffer, shaking back and forth frequently for adequate lysis. After centrifugation at 12,000 rpm for 15 min at 4°C, each supernatant was moved to another pre-cooled Eppendorf tube. Protein concentrations were calculated by the BCA assay kit. In each protein blot analysis, equal amounts of protein (100 μg) from each group were separated by SDS-PAGE and electrotransferred to polyvinylidene difluoride (PVDF) membranes (Millipore, U.S.A.). After being closed with TBST (triple filament buffered saline with 0.05% Tween 20, pH 7.4) with 5% skim milk for 1 h at room temperature, the membranes were incubated with primary antibodies for TXNIP, AMPK and β-actin, respectively, overnight at 4°C. After washing three times with TBST, the membranes were incubated with the corresponding secondary antibodies for 1 h at room temperature. Specific protein bands were observed by applying the ECL kit.

#### Statistical analysis

All statistical analyses were carried out using GraphPad Prism 8.0 software. Results are presented as mean ± standard deviation (SD). Each independent experiment was repeated at least three times to obtain the mean. Differences between the two groups were compared using Student’s *t*-test. One-way ANOVA was used for comparison between groups. *P*<0.05 was considered statistically significant.

## Results

### Screening and collection of SAL targets and CML targets

**Figure 2:**
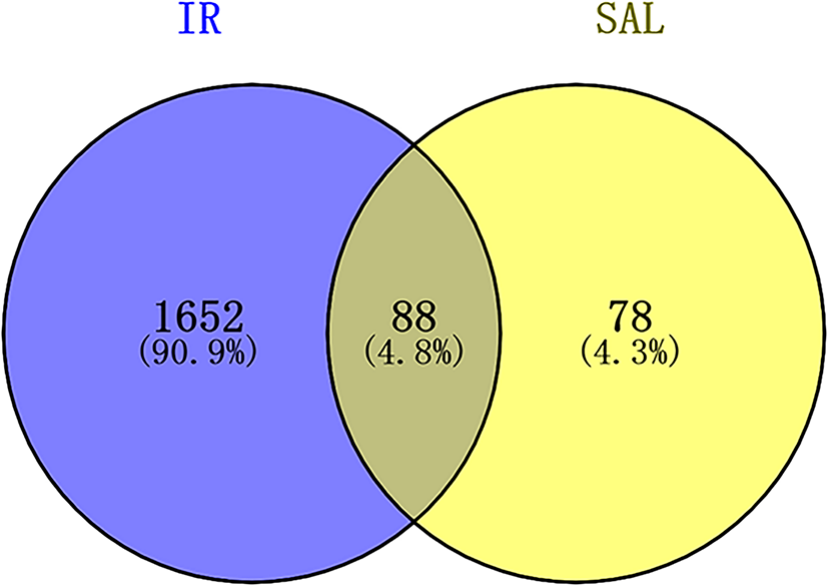
Venn diagram of the gene intersection of SAL and intestinal IR

A sum of 166 targets for SAL were obtained from TCMSP, Swisstarget prediction, and PhammerMap databases. Integrating the disease targets collected from each disease database, a total of 1740 intestine IR injury targets were obtained (Supplementary Table S1). The Venn Diagram was plotted by Venny 2.1, and 88 targets in SAL were found to be potential targets for the treatment of intestine IR injury (Figure 2).

### Construction of PPI network

**Figure 3:**
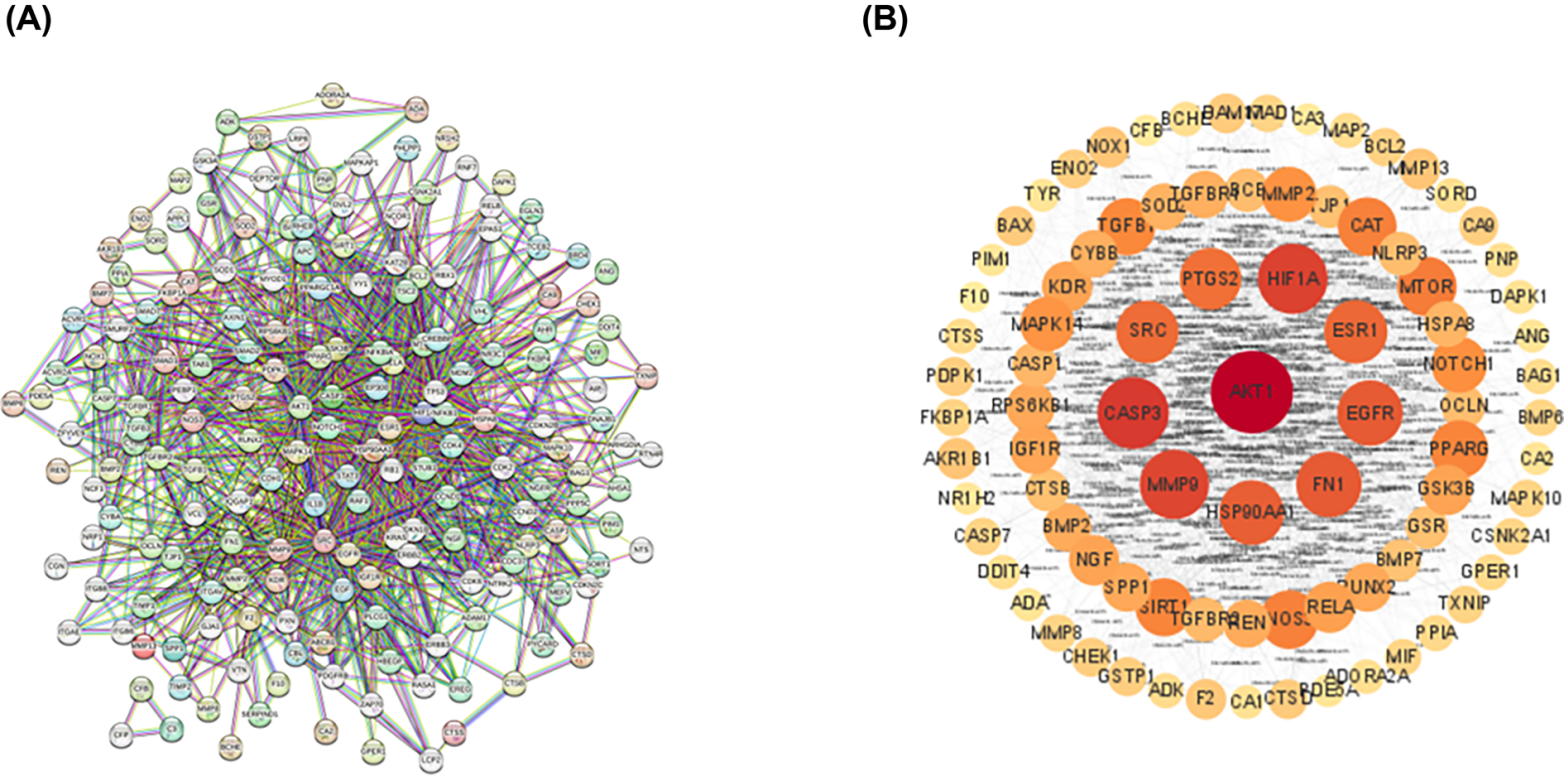
Construction of PPI network and screening of core targets

The interaction targets were channeled into the STRING database, and the network was further visualized and analyzed by Cytoscape 3.8.2 to build a protein interactions map (Figure 3). The nodes in the network diagram stand potential target genes, and the connection between nodes refer to the interaction between proteins. The size and color of nodes are scaled with Degree value. The larger the Degree, the larger the node and the darker the color. The Degree and Closeness Centrality values of each node in the protein network graph were calculated by network analysis method, and a total of 88 cross-target genes were screened, which may be the core target genes for SAL treatment of IR.

### Biological function and pathway enrichment analysis

**Figure 4:**
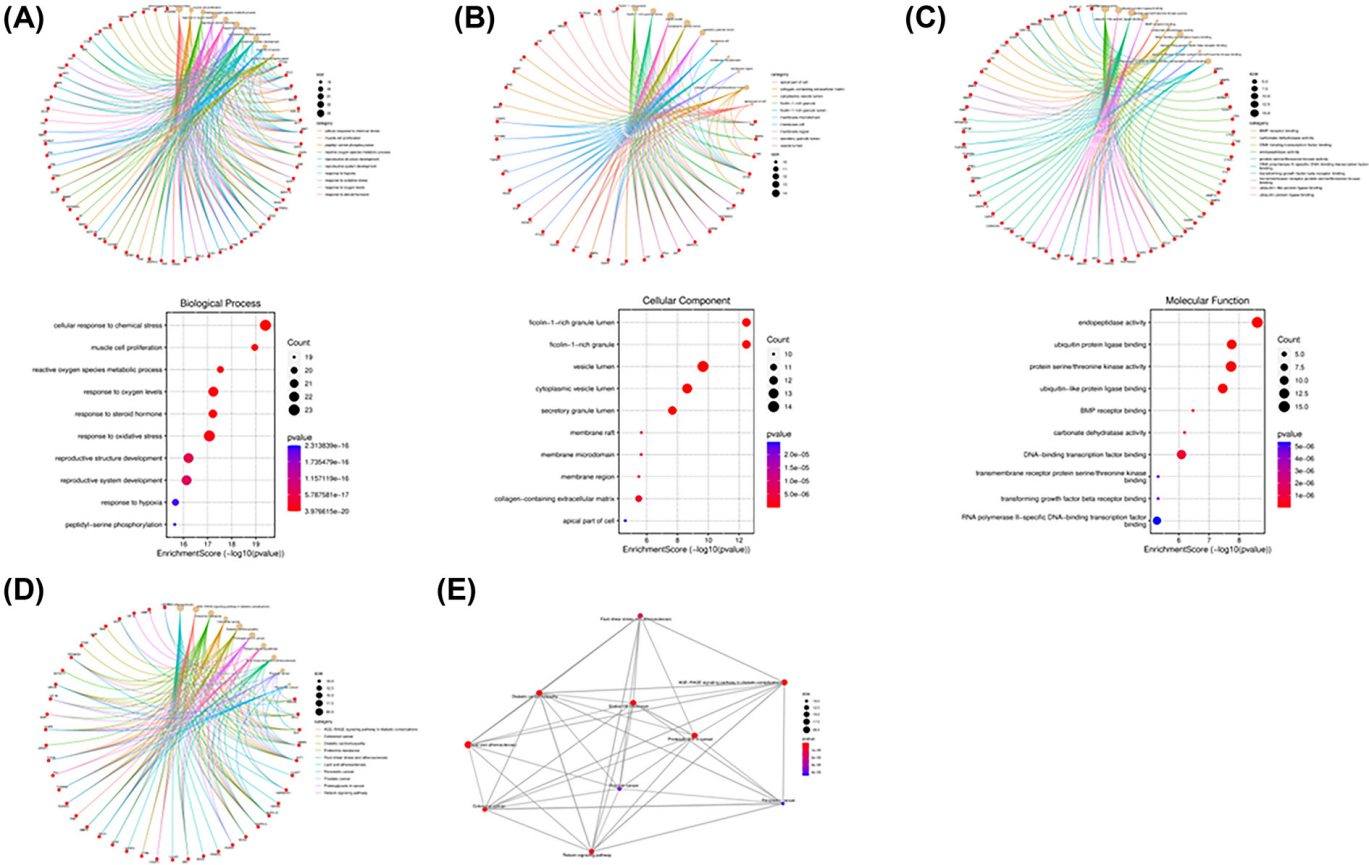
GO and KEGG analysis (**A**) Circle and bubble charts of top ten significantly enriched terms in BPs and related genes. (**B**) Circle and bubble charts of top ten significantly enriched terms in CC and related genes. (**C**) Circle and bubble charts of top ten significantly enriched terms in MF and related genes. (**D**) Circle charts of top ten significantly enriched terms in KEGG pathway related genes. (**E**) The network relationships of important signaling pathways of SAL to improve IR.

To shed light on the multiple biological functions and mechanisms of SAL to alleviate intestinal IR, 88 crossover targets were imported into the DAVID database for GO and KEGG enrichment analysis. GO plot was used to display GO enrichment results including the top 10 items of BP, CC and MF. A grand total of 529 BP (Supplementary Table S2), 59 CC (Supplementary Table S3) and 91 MF (Supplementary Table S4) were acquired using *P*<0.05 as the selection condition. The BP outcomes indicate that the key cross-targets of SAL for the treatment of intestinal IR injury were mainly enriched in apoptotic process, response to hypoxia, regulation of protein phosphorylation, inflammatory response and positive regulation of phosphatidylinositol 3 kinase (PI3K) signaling. The CC results suggest that the potential targets of SAL for improving intestinal IR are mainly focused on the cell surface, components of the plasma membrane and cell junction. The enriched MF terms were mainly enzyme binding, protein serine/threonine kinase activity and ATP binding (Figure 4A–C). With *P*<0.05 as the filtering criterion, a total of 118 KEGG-enriched signaling pathways with 88 targets were retrieved (Figure 4D). The proposed signaling pathways for the treatment of intestinal IR injury with SAL were identified as pathway of different types of cancer, lipid and atherosclerosis, HIF-1 signaling pathway and AMPK signaling pathway, etc. The investigation further analyzed the network relationships of important signaling pathways of SAL to improve IR (Figure 4E). The results showed that there were interconnections between different pathways, and the important active components of SAL were more prone to exert synergistic effects by interfering with distinct signaling pathways to achieve the therapeutic effect of intestine IR injury.

### Building a drug–target–disease network

**Figure 5:**
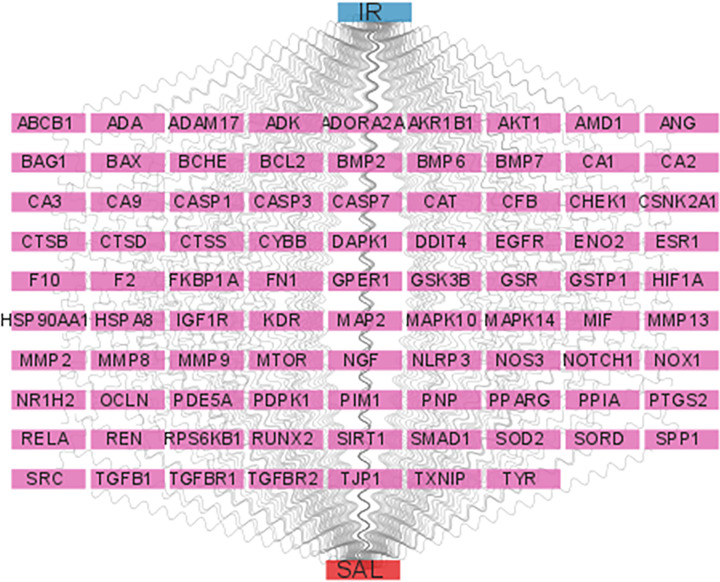
Drug target diseas network The node in blue is IR, nodes in pink are candidate targets of IR and SAL and the node in red is SAL.

We built the SAL-target gene-IR grid to visualize and elaborate the pharmacological potential of SAL to regulate IR. A total of 88 nodes in purple represent common target genes, red nodes represent SAL, and blue nodes represent IR (Figure 5).

### Molecular docking analysis

**Figure 6:**
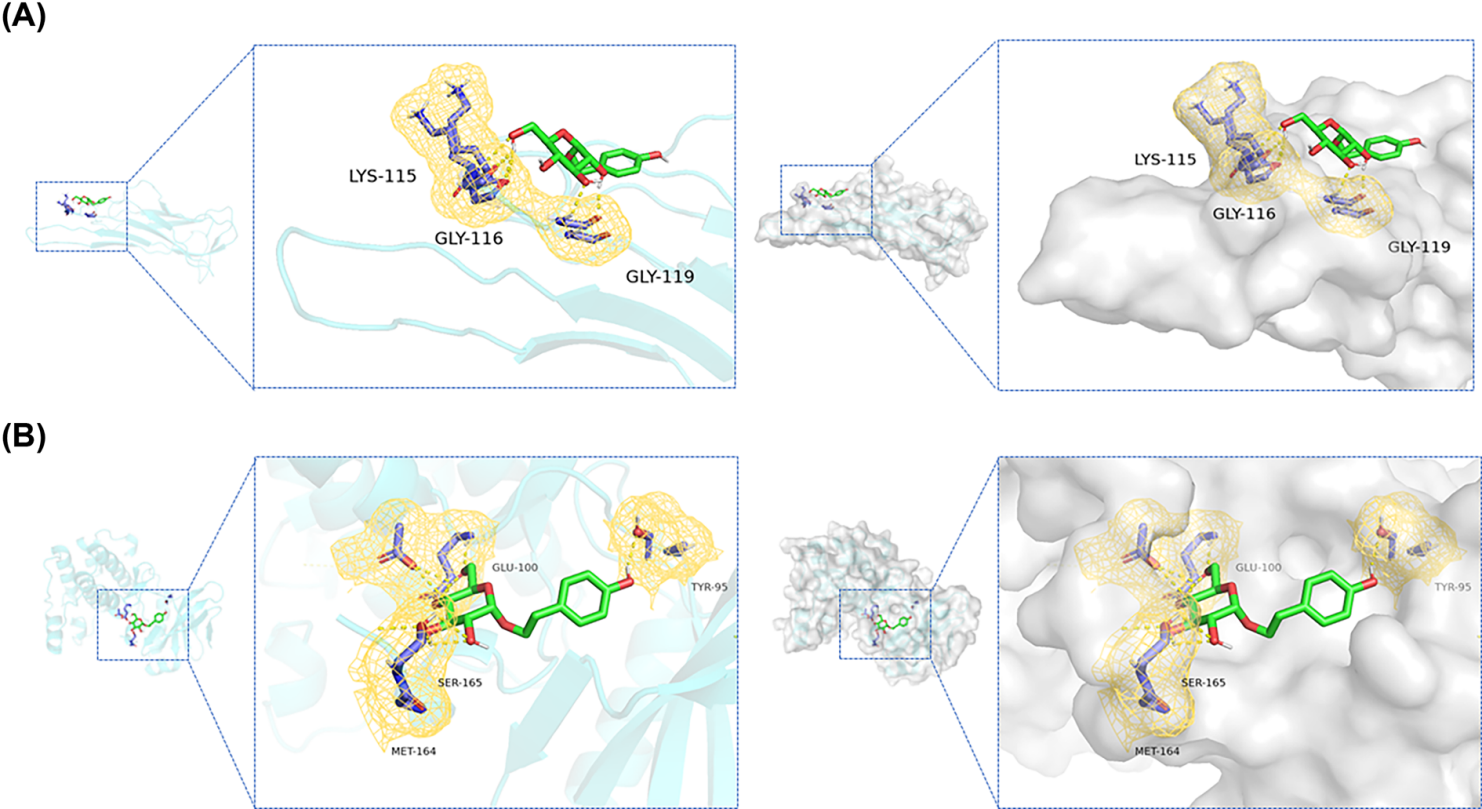
Molecular docking diagram between SAL and TXNIP and AMPK (**A**) Three-dimensional binding mode of TXNIP (PDB: 4GEI). (**B**) Three-dimensional binding mode of AMPK (PDB: 2H6D).

To further explore the binding affinity of the core target to SAL, molecular docking was performed in this study. According to previous findings [24], binding affinity below 0 kJ/mol suggests that the ligand molecule spontaneously binds the receptor protein, while affinity below −5.0 kJ/mol indicates that the ligand molecule has a desirable binding affinity. The molecular docking results showed that SAL exhibited favorable binding affinity for all tested targets with all binding free energies < −5.0 kJ/mol (TXNIP: −6.2 kcal/mol, AMPK: −5.9 kcal/mol). SAL forms hydrogen bonds with TXNIP at LYS-115, GLY-116, GLY-119 and forms hydrogen bonds with AMPK at GLU-100, SER-165, MET-164, TYR-95 (Figure 6).

### Molecular dynamic simulation

**Figure 7:**
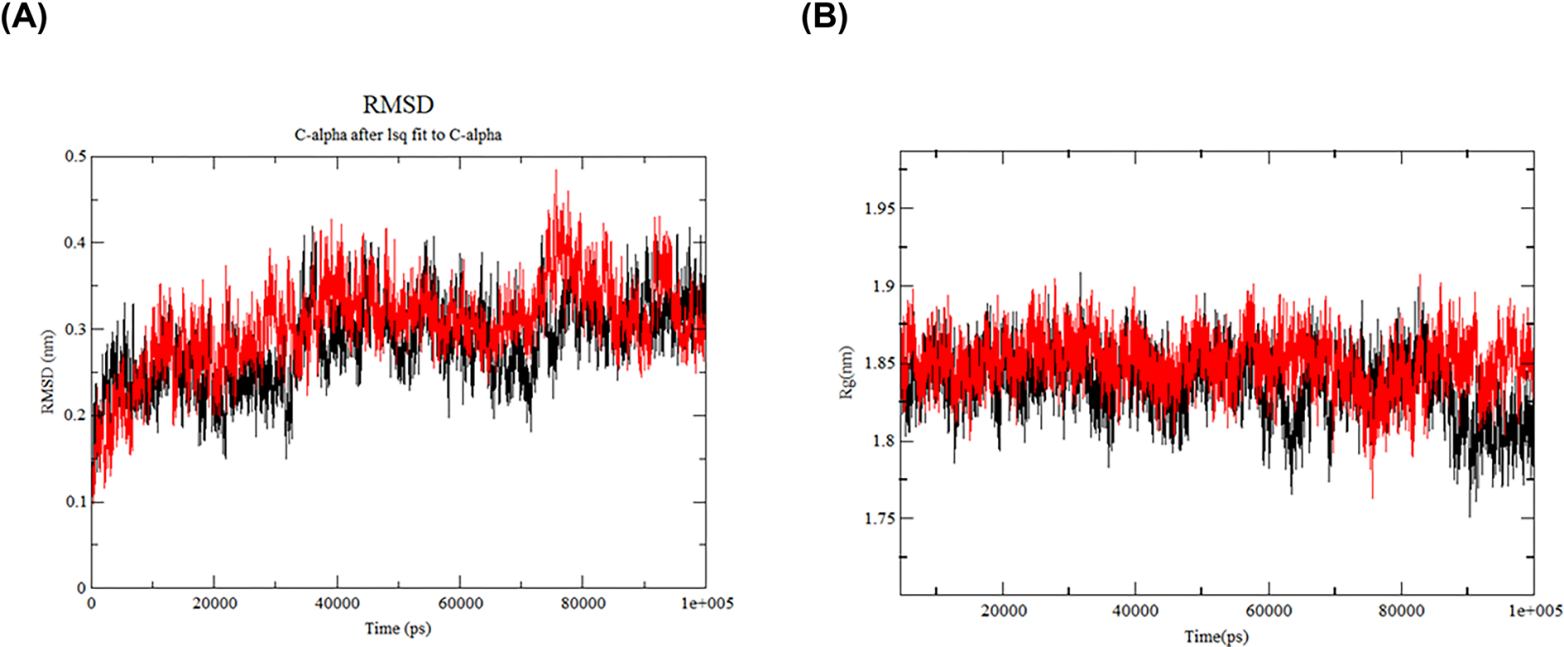
Molecular dynamic simulation between SAL and TXNIP (**A**) RMSD and (**B**). *R*_g_ of SAL-TXNIP complex. Red is the SAL, black is TXNIP.

Root mean square deviation (RMSD) is considered to be a measure of the stability of the system. The lower the average RMSD value, the better the stability of the system. The RMSD values of the free TXNIP and TXNIP-SAL complexes varied during the first 10 ns of simulation and then stabilized after 20 ns. The RMSD values of both remained low and stable during the overall 100 ns simulation (Figure 7A). It shows that after free TXNIP binds the ligand, the complex is fairly stable and the binding does not cause large fluctuations in the structure of the protein. The radius of gyration (*R*_g_) gives an indication of the tightness of the protein structure over the course of the simulation. Analyzing the MD process of free TXNIP and TXNIP-SAL complexes, we found that the *R*_g_ of both was relatively small, and both were less than 1.9 nm, with smooth and small fluctuations, and the protein structure was tighter, demonstrating that the conformation of the complexes did not swell or contract during the simulation (Figure 7B).

### Experimental confirmation of potential targets

#### SAL attenuates intestinal IR-induced histological damage in mice

**Figure 8:**
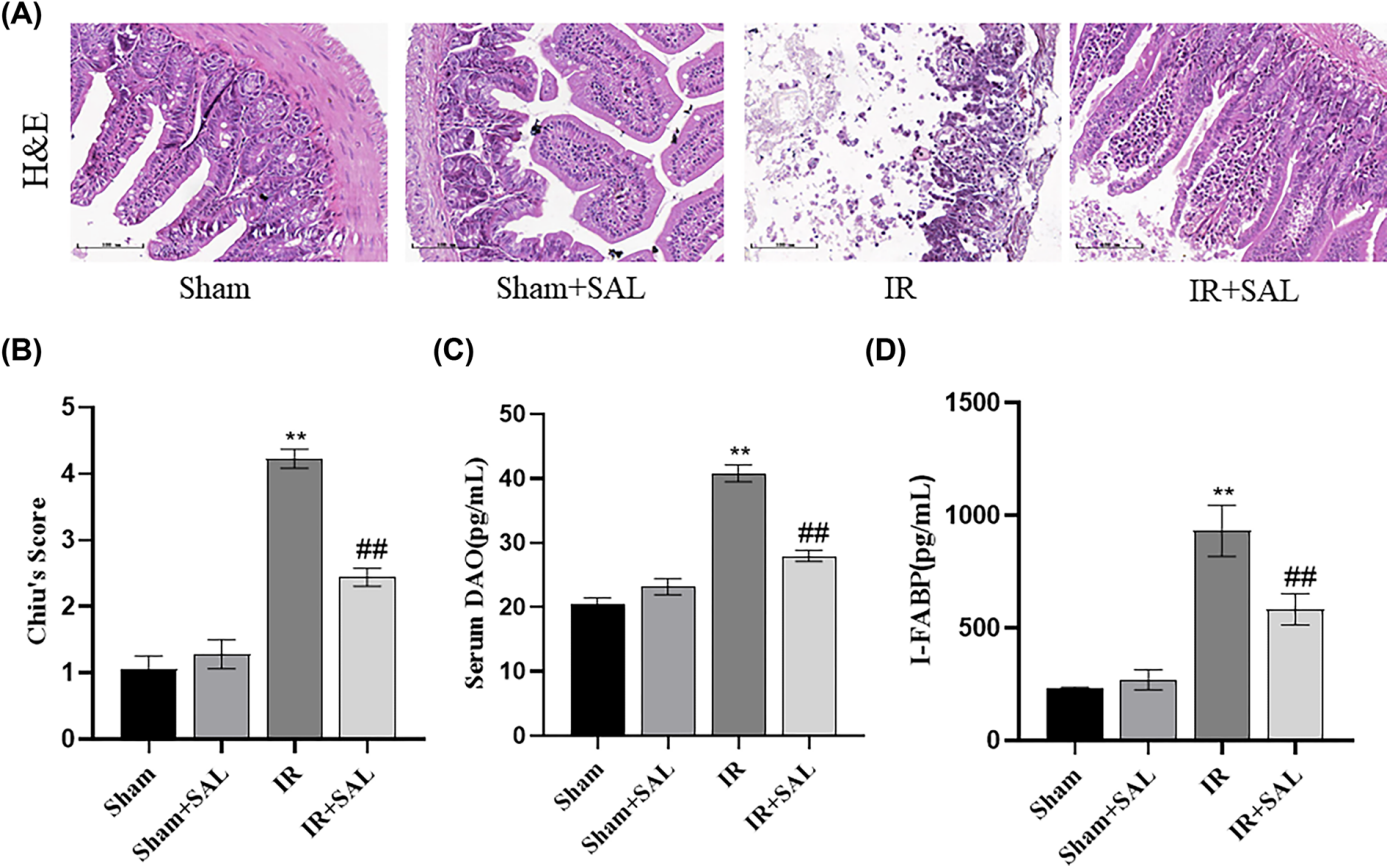
SAL exerts a protective role in intestinal injury *in vivo* (**A**) Pathological results of intestinal tissues of mice in each group (H&E staining, ×200, scale = 100 μm). (**B**) Comparison of Chiu scores of mice in each group. (**C**) Comparison of serum DAO concentration of mice in each group. (**D**) Comparison of serum I-FABP concentration of mice in each group (*n*=6). The results are expressed in mean ± standard deviation. Compared with Sham group, **P<0.01. Compared with IR group, ##*P*<0.01.

To further evaluate the protective effect of SAL on mice intestinal IR, intestinal morphological staining was performed on each group of mice, and intestinal histopathological changes were examined by light microscopy. The results displayed that, compared with Sham group, the intestinal tissue of IR group mice showed disorganized villi arrangement, partially free villi surface, massive necrosis of intestinal epithelial cells, discontinuous structure and obvious destruction (Figure 8A), significantly higher Chiu’s score (Figure 8B), and significantly increased serum DAO (Figure 8C) and I-FABP (Figure 8D) levels. In comparison with IR group, SAL-treated mice exhibited a little quantity of necrosis of intestinal epithelial cells, discontinuous segmental structure of the villi part and no obvious edema were remarkably ameliorated in the SAL-treated mice, and the Chiu’s score was clearly reduced. All these results indicates that treatment with SAL can apparently reduce the pathological damage of small intestine in intestinal IR mice.

#### SAL attenuates the inflammatory response induced by intestinal IR in mice

**Figure 9:**
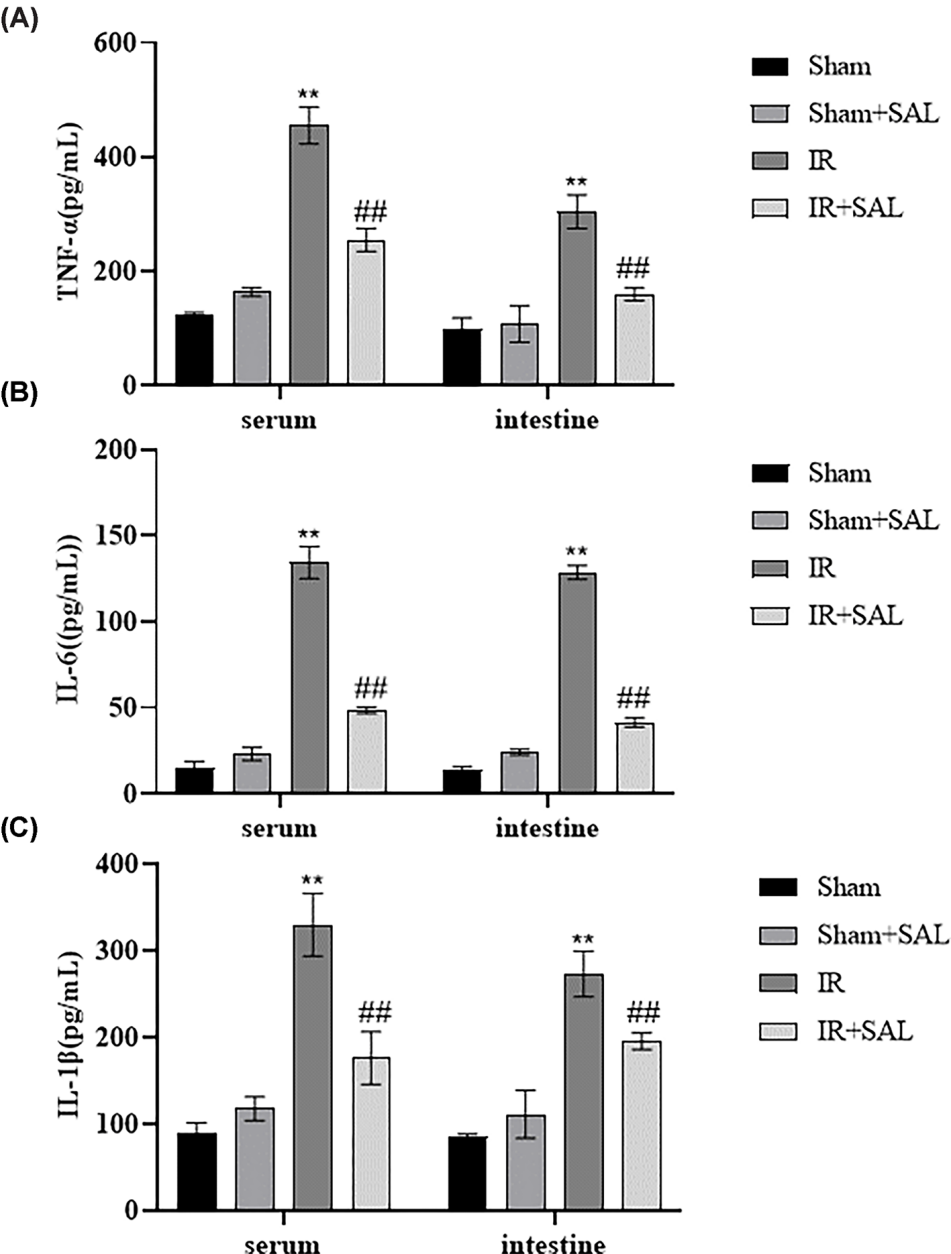
The effect of SAL treatment on intestinal IR inflammation (**A**) TNF in serum and intestinal tissue of mice in each group- α Comparison of concentrations. (**B**) Comparison of IL-6 concentration in serum and intestinal tissue of mice in each group. (**C**) IL-1 in serum and intestinal tissue of mice in each group β comparison of concentrations. *N*=6 for each group. The results are expressed in mean ± standard deviation. Compared with Sham group, **P<0.01, Compared with IR group, ##*P*<0.01.

TNF-α, IL-6 and IL-1β are essential pro-inflammatory factors involved in IR injury. The results of ELISA showed that TNF-α, IL-6 and IL-1β levels were elevated markedly in serum and small intestinal tissues after IR comparison with Sham group (*P*<0.01), and SAL treatment obviously decreased the levels of TNF-α, IL-6 and IL-1β in serum and intestinal tissues after intestinal IR (*P*<0.01) (Figure 9A–C). Thus, it can be concluded that SAL can significantly reduce the inflammatory response induced by intestinal ischemia–reperfusion.

#### Effect of SAL on TXNIP and p-AMPK expression in small intestinal tissues of intestinal IR mice

**Figure 10:**
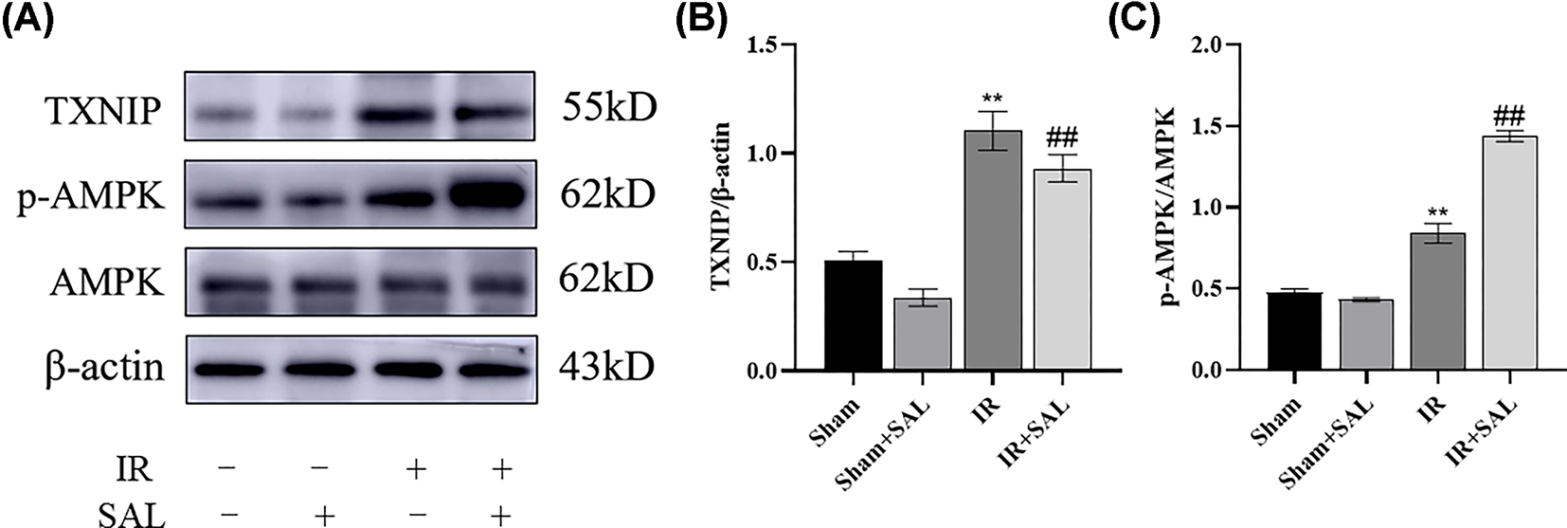
Effect of SAL on the expression of TXNIP and p-AMPK in intestinal tissues of intestinal IR mice (**A**) Expression of TXNIP, AMPK and p-AMPK in mice of each group. β-Acting is used as the loading reference. (**B**) The relative abundance of TXNIP and p-AMPK was measured quantitatively. Compared with Sham group, **P<0.01; ##*P*<0.01 compared with IR group.

PPI network and molecular docking suggest that TXNIP and AMPK are central target genes with high affinity to SAL, and our previous study showed that TXNIP expression was increased in the intestinal IR mouse model [25]. To further confirm the involvement of both in SAL in the treatment of intestinal IR injury and to explore the critical effects of potential central targets of SAL, we semi-quantified the levels of both TXNIP and AMPK target proteins in the intestinal tissues of mice from different experimental groups by protein blotting. The findings revealed that the expression level of TXNIP and p-AMPK was significantly increased in IR mice compared with the Sham group. SAL treatment significantly down-regulated TXNIP protein expression, in contrast, the expression of p-AMPK was up-regulated after SAL treatment compared with the IR group (Figure 10A–C). Accordingly, SAL probably exerts intestine protective effects by activating the AMPK signaling pathway and inhibiting TXNIP expression.

## Discussion

Intestinal IR injury is one of the clinically frequent IR injuries with a very high morbidity and mortality rate. It has been reported that the overall mortality rate in patients with acute mesenteric ischemia is 55.9% [26], and the mortality rate within 30 days in ICU patients is even as high as 71% [27], and MODS and MOF caused by severe intestinal IR are major clinical factors of death in patients with intestinal IR. The pathogenesis of intestinal IR injury is multifactorial, primarily involving inflammatory response, excessive activation and activation of immune cells, oxidative stress, energy imbalance, microvascular dysfunction and calcium overload, etc [28]. Given the complexity of the mechanism of intestinal IR injury, efficient clinical prevention and treatment measures are still limited, and the aggressive pursuit of new therapeutic strategies is a key clinical problem to be solved.

As we all know, Chinese herbal medicine, as an indispensable section of alternative medicine, has been deeply valued by Asian people in clinical practice for its high safety and low side effects [29]. Rhodiola rosea, also known as highland ginseng, has long been considered a valuable medicinal plant for its efficiency in benefiting ventilation and blood circulation, resolving blood stasis, relieving asthma, and improving lung ventilation and blood flow reperfusion [30]. Salidroside (SAL) is a phenolic product isolated from Rhodiola Rosea rhizome, with low toxicity, low side effects and a wide range of pharmacological properties [31]. The results of studies revealed that SAL dramatically decreased the levels of pro-inflammatory cytokines and chemokines such as TNF-α, IL-1β, IL-2, IL-6, IL-8, monocyte chemotactic protein-1 (MCP-1) and MIP-1α in the tissues or serum of MCAO model animals [15,32,33]. Besides, SAL can inhibit the expression of HIF-1α, repress the expression of vascular endothelial contractile factor, facilitate the expression of vascular endothelial diastolic factor, and upregulate the level of vascular endothelial growth factor (VEGF), which is related to angiogenesis during hypoxia, thus improving the vascular endothelial diastolic function [34]. In our study, we successfully established an IR mouse model by ligating the SMA, and observed the intestinal histopathological changes in each group of mice by HE staining, and discovered that the intestinal epithelium of mice in the IR group had obvious changes such as edema and destruction of mucosal villi, inflammatory cell infiltration and increased gap between epithelial cells, and SAL could notably ameliorate such pathological changes in the intestinal tissue of mice. Furthermore, SAL also markedly diminished the expression of diamine oxidase (DAO) and intestinal fatty acid binding protein (I-FABP) in the serum of intestinal IR mice. We are aware that DAO is a highly reactive intracellular enzyme in the cytoplasm of human and all mammalian intestinal mucosal cells, and when intestinal mucosal cells are impaired and necrotic, DAO is released into the blood, leading to increased blood activity, so blood DAO levels are a good indicator for monitoring intestinal mucosal injury [35]. I-FABP is a protein expressed only by enterocytes, which is released into the circulation after enterocyte injury and has been demonstrated to be a promising marker for early identification of intestinal ischemia and injury, all of which are consistent with the results of our study, suggesting that SAL possesses a beneficial therapeutic effect on intestinal IR injury [36].

Inflammation occurs frequently in IR injury and it plays an important role in the pathophysiology of IR injury. Although inflammation is an essential component of the body’s defense system, over-activation of inflammatory cells and secretion of cytokines can lead to severe damage to intestinal epithelial cells [37,38]. The inflammatory response causes the generation of several pro-inflammatory cytokines and chemokines, such as TNF-α, IL-1 and IL-6, which activate and recruit leukocytes. Pro-inflammatory cytokines such as are the major mediators of intestinal barrier dysfunction and damage after intestinal IR injury [39,40]. Therefore, dampening the inflammatory response is possibly an effective way to reduce injury after reperfusion. In the study, we found that the levels of IL-6, IL-1β and TNF-α were markedly increased in the serum and small intestinal tissues of intestinal IR mice, and administration of SAL treatment remarkably declined the expression of inflammatory factors, suggesting that SAL could exert entero-protective effects on intestinal IR injury by attenuating the inflammatory response. However, it is hard to understand the specific pharmacological mechanisms of herbal medicines given the diversity of their components and the complexity of the prescription synergy. Thus, this study combined network pharmacology and experimental validation to systematically investigate the therapeutic mechanisms of SAL on intestinal IR injury, providing novel and unique insights into the treatment of complex diseases such as IR injury with traditional Chinese medicine, and offering new prospects for potential targeted drug therapy for intestinal IR injury.

In the present study, the 88 potential targets of SAL for the treatment of intestinal IR were screened by searching diverse databases and further Venn diagram analysis, which were presented using PPI visual interaction. To clarify the multiple mechanisms of SAL for the treatment of CML, we performed GO and KEGG enrichment analysis of the intersecting targets. Among them, TXNIP, as one of the key molecules of thioredoxin reduction system, serves as a bridge between oxidative stress and inflammatory response, and AMPK signaling pathway, which maintains metabolic stability, were enriched with higher gene number and lower *P*-value in intestinal IR-related signaling pathways, and were strongly associated with other pathways. Importantly, the molecular docking analysis revealed that the docking of SAL with AMPK and TXNIP was < −5.0 kJ/mol, indicating that both possess good affinity for SAL. However, the docking pose of TXNIP seems to be unstable compared with AMPK, and SAL tends to fluctuate in the binding pocket of TXNIP; therefore, we performed SAL and TXNIP molecular dynamics simulations to verify whether such fluctuations would affect the conformation of the bound complex, and it was found that the conformation of the SAL-TXNIP complex did not swell or contract during the simulation.

It is well known that TXNIP is one of the components of the thioredoxin reduction system [41], and its primary role is to inhibit ROS scavenging by binding to the thioredoxin reductase TRX and inhibiting its activity [42]. TXNIP contributes to redox homeostasis, glucose metabolism, inflammation and angiogenesis [41]. Studies have shown that TXNIP knockdown can upregulate the expression of tight junction protein ZO-1 and occluding in intestinal epithelial cells of mice with non-alcoholic steatohepatitis, reduce MPO activity and ROS levels, and reduce hepatitis-induced intestinal mucosal injury [43,44]. We previously demonstrated that TXNIP was increased in both the mouse IR model and the Caco-2 cell oxygenose deprivation/reoxygenation model, and that overexpression of TXNIP exacerbated Caco-2 cell OGDR injury, whereas knockdown of TXNIP attenuated Caco-2 cell OGDR injury, suggesting a critical role for TXNIP in intestinal IR injury [22]. Consistent with present study, TXNIP was obviously raised in the IR group, and treatment with SAL significantly reduced TXNIP, hinting to us whether SAL may also alleviate intestinal IR injury through inhibition of TNXIP expression. However, TXNIP is involved in glucose metabolism and transport, and inhibition of TXNIP induces cell cycle arrest and drives apoptotic events and the consequent potential tumor risk are the main issues in targeting TXNIP applications [45].

AMPK, an AMP-dependent protein kinase, is responsible for regulating cellular energy synthesis and catabolism and are key regulators of cell growth, reproduction and autophagy [46]. AMPK is a serine/threonine-protein kinase heterotrimer complex composed of a catalytic subunit (α) and two regulatory subunits (β and γ), which have different isoforms (α1, α2; β1, β2; γ1, γ2 and γ3) encoded by different genes [47]. The α-subunit comprises a typical serine/threonine kinase structural domain, an autoinhibitory structural domain, an α-junction that acts as an adenosine sensor, and a C-terminal structural domain that interacts with the β-subunit, which contains a serine/threonine-rich loop and a phosphorylation site for the activated protein kinase [48–50]. Being a cellular energy sensor, AMPK is activated under different situations that deplete cellular energy levels, like nutrient deficiency (especially glucose), hypoxia and exposure to toxins that depress the mitochondrial respiratory chain complex [51]. AMPK has been described to reduce IR damage by initiating catabolic processes for ATP production and shutting down anabolic processes for ATP depletion during intestinal IR damage [52]. Interestingly, research has shown that the catalytic α1 subunit of AMPK contributes to the stabilization of intestinal mucosal barrier function, and with the deletion of the AMPK α1 gene, there is extensive tissue structural damage, lipid peroxidation-mediated intestinal permeability damage, and low levels of expression of the ligand proteins occludin and E-cadherin in intestinal IR [53]. Intestinal IR activates macrophages, increases the levels of inflammatory factors such as TNF-α, relies on the AMPK/SIRT1 signaling pathway to suppress inflammatory responses and apoptosis, defends the intestinal endothelial barrier, and effectively reduces intestinal damage caused by intestinal IR [54]. Recent studies have demonstrated that metformin, enkephalin and lipocalin can activate AMPK and thus facilitate recovery from intestinal IR injury [55–57]. Importantly, Zheng et al. [37] et al. found that SAL could abrogate nonalcoholic fatty liver disease through the AMPK-dependent TXNIP / NLRP3 pathway, again consistent with the results *in vivo* validation experiments in our study, where AMPK was activated in the mouse IR model group, and after SAL treatment AMPK was further activated. However, Qin et al. [58] discovered that p-AMPK activity was reduced in mice undergoing myocardial IR. We speculate this might be due to the fact that redox and metabolism have been imbalanced in mice after 45 min of ischemia and 24 h of prolonged perfusion, whereas in our study, we showed elevated AMPK expression at 2 h of reperfusion when we established a mouse model of intestinal ischemia–reperfusion, suggesting that AMPK at this time can produce an adaptive response early during IR, which is consistent with the study of Yu et al. [59] in which P-AMPK expression was elevated early in sepsis. Hence, the results of the present study suggest that SAL is capable to alleviating the intestinal IR-induced inflammatory response and thus exerting protective effects by acting on TXNIP and AMPK, but whether it acts on both separately or whether SAL affects the interaction of TNXIP and AMPK is not yet known, which is one of our future research objectives.

## Conclusions

In summary, the present study combined network pharmacology and in vivo experimental validation to predict the effective mechanism and main targets of SAL for the treatment of intestinal IR injury, and validated the predicted results by molecular docking, molecular dynamics simulation and *in vivo* experiments, suggesting that SAL has the ability to act simultaneously on multiple targets and signaling pathways to produce synergistic effects, and is an effective means to treat intestinal IR injury. Our study indicates that SAL may affect intestinal IR injury by regulating the expression of TXNIP and AMPK, and these molecules have important roles in regulating inflammatory responses and energy metabolism. Consequently, we could conclude that SAL might be an effective therapeutic agent for intestinal ischemia–reperfusion injury, but further experiments are still needed to explore its mechanism.

## Supplementary Material

Supplementary Figures S1-S4 and Tables S1-S4

## Data Availability

I confirm that all original raw data is available at the time of submission. As per the Data Policy, these data will be stored for a minimum of 10 years and will be made available to the Editorial Office, Editors and readers upon request. I confirm that, if present in the manuscript, the full uncropped and unedited versions of Westerns blots with molecular mass markers are included as a separate supplementary file accompanying the submission. I confirm that all images accurately and faithfully reflect and represent the original data, no ‘beautification’ of the images in the final figures has taken place. Any modification of figures has been clearly stated in the figure legend. I confirm that all data presented in this submission were generated by the authors, at their affiliated institution(s) at the time of submission. Any data obtained from a public repository is explicitly stated as such, along with access details, in the article file.

## Abbreviations

BP: biological processes
CTD: comparative toxicogenomics database
CC: cellular components
DAO: diamine oxidase
H&E: hematoxylin and eosin
I-FABP: intestinal fatty acid binding protein
IL: interleukin
IR: ischemia–reperfusion
MCP-1: monocyte chemotactic protein-1
MF: molecular functions
PI3K: phosphatidylinositol 3 kinase
PPI: protein–protein interaction
RMSD: root mean square deviation
SAL: salidroside
SMA: superior mesenteric artery
TCMSP: Traditional Chinese Medicine Systematic Pharmacology
TNF: tumor necrosis factor
VEGF: vascular endothelial growth factor
