# Supplementary material for: Network pharmacology analysis combined with experimental validation to explore the therapeutic mechanism of salidroside on intestine ischemia reperfusion
Source: Biosci Rep. 2023 Aug 25;43(8):BSR20230539. doi: 10.1042/BSR20230539 (PMC10462912; doi:10.1042/BSR20230539)
Supplement: Supplementary Figures S1-S4 and Tables S1-S4 [file BSR-2023-0539_supp.pdf]

Dear editors, thank you for your careful review and constructive suggestions regarding our manuscript. We have revised the manuscript inaccordance with the comments.

1. Regarding supplication an ORCID for the corresponding authors account, we have added the ORCID into the Manuscript Comments box.
2. We have ensured that your Supplementary Figures/Tables include a title and/or a legend.
3. Regarding the Data Availability Statement , we have amend the statement in the submission file to match the statement provided in the manuscript.
4. Regarding the full uncropped and unedited versions, we have provided raw WB-ready data as follows:
   The lanes from left to right: Sham, Sham+SAL, IR, IR+SAL

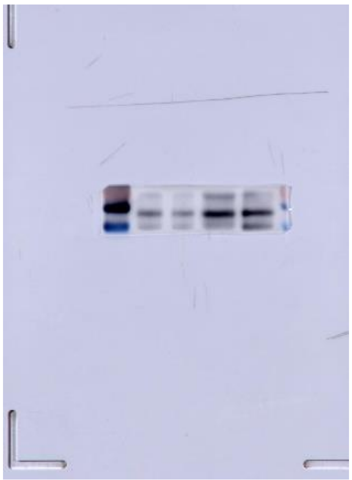

Supplementary Figures 1：TXNIP, 55kD

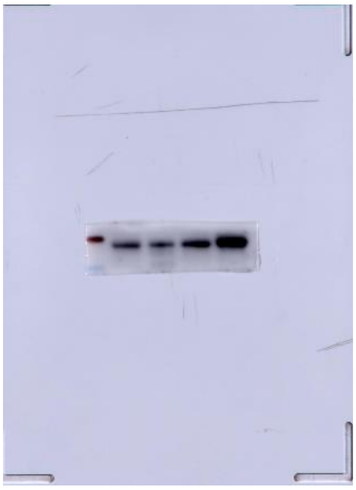

Supplementary Figures 2：p-AMPK, 62kD

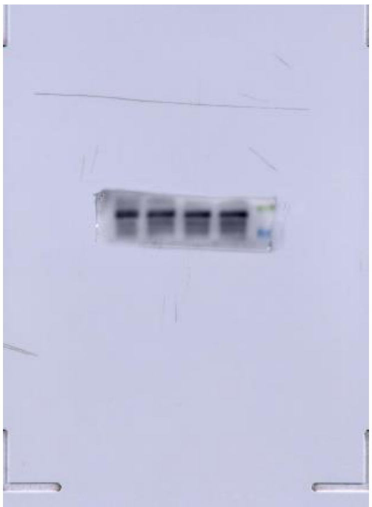

Supplementary Figures 3： AMPK, 62kD

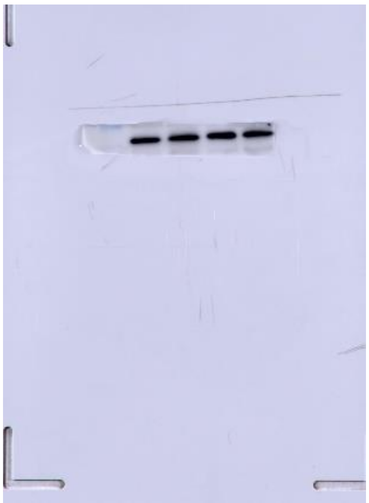

Supplementary Figures 4： β-actin, 45kD

Supplementary Table 1 Target genes of IR and SAL

Target of SAL	Target of IR
TYR	TP53
CA2	CTNNB1
CA7	PIK3CA
CA1	IL6
CA6	VIP
CA12	SMAD4
CA9	TNF
CA4	IL10
CA5A	FLNA
CA14	FABP2
CA3	MIR21
ADA	NOD2
GSK3B	IL1B
ADK	VEGFA
ADORA2A	TLR4
PNP	EDNRB
AKR1C3	PTEN
P2RX3	ALB
CDA	EPCAM

GPER1	SI
PTGS2	AKT1
MBLAC2	CRP
BCL2	CXCL8
BAX	STK11
MMP2	HMOX1
MMP9	GDNF
ABO	CTLA4
ADAM17	MPO
ADH1B	ICAM1
AKR1B1	MUTYH
AKT1	HIF1A
AMD1	GCG
AMY1A	ACE
AMY1B	CASP3
AMY1C	NOS2
AMY2A	SOD1
ANG	KIT
AR	TGFB1
ARF1	IL1RN
AURKA	SLC5A1
AZGP1	CFTR

B3GAT1	TLR2
BACE1	ABCB1
BAG1	EDN1
BCHE	XDH
BMP7	EGFR
CA1	BAX
CA12	SLC9A3
CA2	MIR145
CASP7	ECE1
CCNA2	ATP7A
CDK2	JAK2
CFB	CCL2
CFD	MIR126
CHEK1	FAS
CMA1	CDKN2A
CRAT	F2
CSNK1G2	SST
CSNK2A1	IFNG
CTSB	NOS3
CTSD	FGFR2
CTSS	INS
CTSV	APOB

DAPK1	PPARG
DDX6	STAT3
DHFR	BRCA2
EGFR	MIR192
EPHB4	SELP
ESR1	SRC
ESR2	EPO
ESRRG	BCL2
F10	PTGS2
F2	F5
FAP	CALR
FCAR	HSPA4
FKBP1A	TNFRSF1A
GALK1	TGFBR2
GBA	MGAM
GPI	EGF
GSR	F3
GSTP1	ENG
HCK	SERPINC1
HK1	VWF
HSP90AA1	TEK
HSPA8	CUBN

IGF1R	CD34
IGLV2-8	PLG
IMPA1	SOD2
KDR	MIR17
KIF11	PLAT
LGALS7	CCND1
LGALS7B	MIR34A
MAPK10	CXCL12
MAPK14	MAPK14
MIF	MIR143
MMP13	MAPK1
MMP8	IL17A
MTAP	SDHB
NOS3	APOA1
NR1H2	IL4
NUDT9	GPT
PAH	STAT1
PDE4B	PLA2G2A
PDE4D	JUN
PDE5A	GAST
PDPK1	NPY
PGR	IRF1

PIM1	NOS1
PLK1	APP
PNP	SELE
PPARG	CCK
PPIA	MB
PYGL	XIAP
QPCT	MIR155
REN	IL18
RTN4R	CALCA
SHBG	NPPB
SOD2	NFE2L2
SORD	FGF2
SRC	PIK3C2A
TGFBR1	CDKN1A
TGFBR2	FBXW7
TTR	ELANE
TYMP	MYC
TYMS	CYCS
YARS1	SERPINA1
ABCB1	HMGB1
ABCB11	YRDC
AKT1	ODC1

ALPL	ATM
BAX	TJP1
BCL2	KNG1
BMP2	MMP9
BMP6	SMAD2
BMP7	KDR
CASP1	HGF
CASP3	MIR210
CAT	MTOR
CDH2	ADCYAP1
CERT1	CAT
CLDN11	ENO2
CYBB	IL2
CYP7A1	MIF
DDIT4	S100B
ENO2	NPPA
FGF15	CD55
FN1	CXCR4
HES1	MTHFR
HIF1A	MIR221
MAP2	FOS
MTOR	CD40LG

NGF	SLC9A1
NLRP3	IGF1
NOG	COL3A1
NOTCH1	IGF2
NOX1	BDNF
NR0B2	ACTA2
NR1H4	EGR1
OCLN	MYD88
PRKAR2B	B2M
PYCARD	GFAP
RELA	PON1
RPS6KB1	ADA
RUNX2	KCNJ5
SIRT1	RIPK1
SMAD1	THBD
SMAD5	ITGB2
SMAD9	MMP2
SOD2	MAPK8
SP7	CASP9
SPP1	PIK3R1
TGFB1	MIR122
TJP1	SETD2

TUBB3	MT-CO1
TXNIP	HSPB1
	BRCA1
	HSPA8
	ITGAM
	MIR140
	GREM1
	EDNRA
	CHGA
	MAP2K1
	MIR141
	FLT1
	PARP1
	AOC1
	NFKB1
	TXN
	CASP1
	GHRL
	LEP
	DPP4
	IL12A
	VCAM1

GPR31

ADM

TRPM7

HSPA1A

NGF

HP

CREB1

ALOX5

PECAM1

SELL

MIR222

SERPINA3

PPARA

C4A

IL13

ACTB

ANO1

PRKAA2

ANGPT1

IL1A

NTS

SLC6A19

GJA1

IKBKG

LMNA

MIR146A

ADORA1

KITLG

SLC12A2

SLC17A5

MUC1

MDM2

ABCG2

PRKCE

NLRP3

CSF3

ADCY10

IL5

ADAMTS13

CD40

GP1BA

TGM2

HAMP

MBL2

PIK3CG

AGTR1

MIR106B

CD36

RHOA

FABP3

ITGB3

ESR1

PTGS1

LCN2

LPL

LRP2

HSPA5

MIR27A

CDH5

LTF

CXCL2

CYP2C19

ADAMTS3

TNFAIP3

SMAD7

CYBB

SDHA

MST1

BMP6

CEACAM5

RELA

HBEGF

FABP1

ACE2

CBS

CCN2

CCL5

F13A1

CD274

CCR5

ADAM17

TAC1

MIR15B

MIR25

CXCL10

FASLG

ABCA1

PLA2G4A

GAPDH

DAPK1

PTPRC

HSPG2

NOTCH1

SMARCAL1

MCM6

KCNQ1

GATA4

SLC8A1

GAL

FN1

IL22

ITGB1

BMP2

PRKCD

CEACAM3

CX3CR1

CAV1

CP

CTSL

MIR142

ITGA6

REN

THBS1

MPL

PTPN11

IL15

MME

SLC9A3R2

ACHE

IL9

BCL2L1

SERPINE1

MIR29B1

GSK3B

SMARCA4

TIMP1

COL1A1

VIL1

RAC1

MIR203A

ITLN1

LGALS3

MMP1

IL33

MAP2

IL7

IL37

SLC2A1

SIRT1

CSF2

SH2B3

PTK2

CCL3

NOTCH2

NT5E

MIR342

MCU

G6PC1

SLC3A2

CYBA

COMT

GSTP1

MIR451A

ACTC1

IL2RA

FGF7

SHH

CIITA

ATG16L1

CASP8

PDE5A

RAF1

TGFA

CEACAM1

AGT

CD14

CDKN3

PCNA

SGK1

OCLN

CTSB

MALAT1

MAPK3

ADIPOQ

SPP1

SELPLG

MIR30A

GDF15

BLVRB

HTR2A

TOLLIP

ANGPT2

HSPD1

MIR195

YAP1

NCF1

NOD1

TNNT2

FABP12

SLC11A2

CXCL1

CXCR2

SERPING1

APOH

COX5A

GCLC

MIR29A

EPRS1

CD44

THPO

SLC1A2

NEAT1

LOX

TERT

ELAVL1

TF

C3

GSR

NTRK1

F12

PRKCA

FGF1

PRF1

GZMB

SLC22A4

KRT18

LDLR

CD80

HRH2

VEGFC

PTK2B

IDH1

S100A9

SLC40A1

ARID1B

HSP90AA1

DNAH8

MMP14

CLU

H19

CASR

LTA

RUNX1

MIR15A

C1S

AIFM1

NR1H2

GLP2R

TIMP2

EZH2

CHAT

CYP2C8

AGTR2

SOCS3

ALDH2

SDC1

SLC6A4

ASIC1

HMGCR

PDP1

AGER

SLC9A2

XBP1

TGFB2

ADRB2

IGFBP3

ADORA3

PLA2G6

IL23A

SLC16A1

TGIF1

MAPK8IP1

SCARB1

LONP1

PRKCZ

NFKBIA

GHRH

ANXA5

IRF5

MIR214

FGA

SHC1

COL4A1

GRP

UCN

CKB

DDIT3

JAK1

DES

SP1

F2R

TRAF6

SCN5A

PROM1

EIF2AK3

PCSK9

BAK1

BECN1

ITGA4

ADRB1

NAGLU

GH1

EPAS1

TNNI3

IL1R1

ATP7B

IGFBP2

CYP2C9

CDKN2B-AS1

TIGAR

NOL3

IL6R

MIR24-1

CST3

MIR144

UGT1A1

ITGA2B

PLAU

MIR211

IGF1R

ADORA2B

PPIG

AKR1B1

TYK2

CRH

SLC5A6

ADORA2A

CFLAR

S100A8

CHRM3

TNFRSF1B

NCAM1

MIR486-1

SLC22A2

TSC2

RMRP

CHUK

GSTM3

PRKCB

CSF1

KMT2D

IDO1

SLCO2A1

GRK2

IQGAP1

CTSD

TFRC

PLA2G7

TFF1

NQO1

GLUL

MAP3K7

AVP

TNFRSF11B

STING1

MIR125A

CNTF

MEG3

GUSB

NGB

KEAP1

HMOX2

MIR10A

IRS1

GGT1

MYH7

ERN1

HTR3A

APOC3

MIR324

PTGER4

IL11

BAD

FGF23

CXCR3

BCHE

EPX

ENTPD1

MIR204

MIR199A1

IL18BP

COL14A1

MYH6

MIR23A

NRG1

POMC

NTF3

ABL1

FRAS1

CASP2

STAT5B

SLC22A3

BMP7

RETN

TRAF2

IKZF1

BMP4

ANXA2

MAPK10

MIR200A

BIRC5

DCN

SOD3

GSTM1

MIR133A1

NR3C1

ARG1

SLC8A2

OLR1

CD69

TSPO

CCL20

MIR193A

IL2RB

CYP2J2

KCNJ11

NR3C2

TKT

CYP27B1

MIR124-1

NEU1

MIR320A

CHKA

RIPK2

ATF6

MIR92A1

CDK5

APEX1

GBA3

F2RL1

AIF1

PDGFB

TGFBR1

LIF

F11R

ADNP

CPOX

MIR532

MT-CYB

CX3CL1

C1R

BRD4

KCNMA1

ITGAL

SERPINF1

ATF3

TIMP3

MIR125B1

IL6ST

NES

MYBPC3

LGALS1

AREG

ERCC2

NR1H3

EIF2S1

SPTAN1

CD46

TNFRSF10B

FOXC1

CASP7

CD86

PLA2G1B

MIR26A1

FOXO3

HNF1A

SAA1

EPHX2

CAST

AOC3

PLAUR

GLS

MIR106A

FASN

FOXM1

CAMK2G

TNFRSF10A

MIR485

TNFSF10

DUSP1

SULT1A3

IL3

IL1RAPL2

MAPT

GPBAR1

HTR2B

AQP4

INSR

FYN

LDHA

HCRT

SLC27A4

KCNQ1OT1

ACP1

CTSG

GRIN2B

BAG1

ATF2

OGG1

OPRM1

ACVR2B

BID

NR4A2

REG3A

SLC18A3

ANXA1

PRKG1

PTX3

TRPV4

MAP3K5

F10

C5AR1

PTGIS

ENO1

DNM1L

PTAFR

PTPN3

ABCC8

RNASE3

ABCG1

TRPV1

ACTG1

GOT2

PER2

SERPINF2

CHI3L1

BDKRB2

CYP1B1

NTN1

MAOA

PF4

IL1RL1

STAT5A

AIM2

FGB

AQP1

PPARD

MIR378A

MBP

SNAI1

PPARGC1A

WNT4

GAS5

MICB

HYOU1

IL7R

MIRLET7B

CXCR1

MAP3K11

PIK3CB

UCP2

APLN

E2F1

BSG

MAPK9

MEF2C

MASP2

HOTAIR

XRCC1

PXN

FFAR2

CA1

ALOX12

SLPI

DRD2

RIPK3

TACR1

HSP90B1

PROC

ICAM2

PRKAA1

NRP1

RPS6KB1

MIR455

PINK1

CXCL16

CYSLTR2

P2RY12

MICA

ATP1A1

EGR2

SERPINA4

FOXP2

NOX4

TPI1

TYR

C5

PROCR

VEGFB

EPHA2

RYR1

LRP6

MAP2K3

CCR2

NPR3

BCL2L11

PDE4A

GPX1

GC

FOXO1

HDAC1

VTN

PLA2G10

SMPD1

NGFR

FHL2

CTF1

COL4A2

CYSLTR1

EFNA1

PPIF

MIR424

G6PD

CASP6

CDC42

CCL26

PARK7

ITGA7

PPP1R1B

GJB1

CRHR2

THY1

CR1

CFH

CACNA1C

DIABLO

WT1

OXA1L

SAT1

AHSP

RPS27A

COL7A1

UGCG

GFER

ITGA1

THBS2

MIR139

KLRK1

JMJD1C

UTS2

MIR499A

EPHA3

MIR19A

KL

CEBPA

CBL

MMP13

KRIT1

NAMPT

PTGER3

H2AX

IL15RA

P4HB

MAP2K4

OPRK1

TNFSF12

TGM1

VLDLR

CXCL5

GPR65

CNR1

THRA

BNIP3

NDUFA13

MIR503

PAPPA

GCK

SLC29A1

EIF4EBP1

PTPA

PSAP

BCAR1

CLDN5

SLC1A1

ATF4

PEPD

MAPKAPK2

FGF19

OPRD1

MIR130B

MTA1

LY96

TNC

BCL2L14

MT-CO3

WNT3A

PDIA2

MAP3K14

PTGES

MIAT

LTB4R

BEST1

IRF2

BLOC1S1

SNHG15

SLC5A2

MAPK11

CYP2B6

PDPK1

ADPRH

IFNB1

CD47

TTN

ITGAV

MIR675

MIR186

SELENOP

HTRA2

FADD

GPX3

SYNGAP1

C3AR1

CXCL9

PTGER2

GCLM

NPY4R

PDE3A

MAP3K1

TUBA1B

NLRP1

TNXB

MAP2K6

MIR208A

PRKAG2

DLG4

BPI

NOX1

DAXX

MSRA

SENP3

LBP

C4B

S1PR1

KCNN3

NFAT5

NTRK2

IL18RAP

CDON

SOAT1

SLC4A4

UNC5B

CAPN2

SLC1A3

HABP2

CRK

HSF1

VCP

MAOB

SNAP25

ABRAXAS2

AXL

DUSP6

S100A6

PVT1

EPOR

PDGFA

JUP

CCL22

NPPC

SNHG16

GRIN2A

PRKCI

LAMA1

MIR24-2

ABAT

NDRG4

SLC2A3

RHOB

ABCC9

ATP2A2

KCNH2

ZC3H12A

POSTN

SNCA

MAP2K7

LEPR

SP3

WDR26

YY1

RUNX2

MRE11

ITCH

CKM

SLIT2

CSF1R

MMP8

IRAK1

IGFBP4

MIR191

NMT1

SIRPA

GNA11

CD59

LAMB1

IL18R1

IRS2

CNR2

AMPD3

HRH1

MEF2A

ACACA

SALL1

GADD45A

GLUD1

SYVN1

GLO1

SLC24A3

HLA-G

FADS1

CPT1A

PANX1

TMSB4X

DPYSL2

BIRC2

OSM

P2RY1

VDAC1

TRAF3

VCAN

GAD1

TINAGL1

TAB2

TCAP

ADK

SLC22A6

ITPR1

HNF1B

FPR1

LATS2

AMD1

ADM2

MIR130A

HSPB2

NR4A1

KLK1

CCNL2

PPIA

FBXO32

COL18A1

ABCB7

CD58

KLF3

MIR98

TLR7

CCN1

FCGR3A

IGFBP5

AHSG

AMPD1

BTG2

APAF1

FMR1

GNAI1

CSK

CYGB

FOXC2

ROCK1

PDIA4

TRAP1

FCGR3B

NLRX1

PRKAB1

TAB1

SLC25A4

APOM

GLRX

MIR27B

GJC1

SNHG1

GSDMD

ATP1A2

SPARC

MIR101-1

ACSL4

CHEK1

ANGPTL4

HPRT1

PRDX5

TRPC6

PDYN

MT1A

CUL3

SIAH2

CXCL11

CAPN1

RNF5

BMI1

S100P

IL16

VHL

PIAS1

TFG

COL6A1

EPHX1

RHOD

HCRTR1

CLCN3

SLC6A3

LAP3

CA9

PSEN1

PTGDS

PTPRU

INA

GPR39

MIR30C1

CHRNA7

ZFAS1

LAMA2

ROCK2

PTGIR

FKBP1A

BTK

LINGO1

RAP2C

TNIP1

ACVR1B

PLSCR3

TRIM28

PRMT5

ALDH1A1

PRSS2

ADAM10

C1QTNF3

BYSL

CXCL13

SCG2

CS

UCA1

IL1R2

SLC9A8

APLNR

PNOC

GSTA2

SLC29A4

HSPB6

ATP2B4

YTHDF1

FGF4

CPT2

AQP9

ACTN4

SORD

DDIT4

MERTK

NAXE

ALDH9A1

DEFA1

LAMA4

BDKRB1

SPON1

MIR370

VSIG4

MANF

EFS

HMCN1

PRKN

LAMB2

ZFYVE9

TRIM45

MLYCD

MYDGF

MIR874

RTN4

TXNIP

CLCN2

YWHAG

NID1

FABP4

HK2

ADAMTS9

SIRT3

POR

MIR1-1

MIR449A

TBXA2R

PRDX6

IFIT3

TXN2

PLSCR1

FBLN2

ADCY1

PRNP

MSTN

SMAD1

PROKR1

PPP1CA

MEF2D

SRPK1

PRKCG

NPR1

BCL2L15

DPT

MSX2

GRIN1

CTSS

MIR433

TNFSF11

PNP

MLKL

TP53BP2

SMURF2

F8

NORAD

DUSP19

MIR26B

SMPD3

CBR1

CREB3L1

CLEC3B

TIE1

CIRBP

SART1

BLZF1

SIVA1

SIRT2

MIR188

HSPE1

TIAL1

NDUFB2

COL6A2

HSPA14

AGGF1

MAP3K12

CAMK2A

TAT

FNDC3B

LAMC1

SELENBP1

PPP2CA

TFPI

SLK

CPB2

TGM3

CD38

GRIK1

MIR107

COL6A3

S1PR2

GPNMB

CD74

YWHAZ

SNHG14

SNHG12

SFN

MIR181A2

PPP2CB

TNFRSF9

PRELP

ALOX15

MCAM

ANGPTL2

TNFRSF12A

GPR55

HMGB2

RPS6KA5

MIR92A2

LRG1

CSNK2A1

TRPM2

CD163

PNPO

FKBP4

HADHA

PTGER1

CALM2

GLRX2

RCAN1

F11

FPR2

GSDME

TGFBI

TNIP3

SCGB1A1

MARCKS

IRF9

NEFL

CRYAB

KCNJ8

ATG4B

AK1

TNFAIP2

TREM1

YWHAB

XIST

KDM3A

ROBO1

PPIC

KCNN1

YBX1

MSRB1

AQP8

MLC1

RBMS1

ESM1

CABIN1

SIRT6

CALCRL

APOD

KCNJ1

CAV3

COX4I1

FRZB

NCK1

ALDOA

CBX7

EEF2

MMP10

CALM1

AGPAT1

MIR381

TIPARP

AGRN

CA3

CHRFAM7A

HSPA1B

METTL14

TPH1

FGD5-AS1

PROK2

EIF4E

OSMR

EIF4A2

FXYD1

SOS1

MATN2

PSMC6

SERPINI1

SLC6A9

VPS28

CASP4

RAE1

EED

HADHB

WWP2

MAPKAPK3

ATP5F1E

CXCL6

EMILIN3

PPP1R15A

TOMM40

WNT9A

AIMP1

SORBS3

MAP4K2

TRIM27

MAS1

DDAH1

RYR2

AOX1

GSS

USP49

HSPA12B

DIAPH1

ERBB4

EVA1A

ALDH5A1

COL15A1

GPR4

LUM

SMPD2

EIF4G1

SIK2

PTHLH

NEK8

PRKACA

MARCKSL1

COMMD5

CREB3

RGS19

MIR4435-2HG

MAFB

MIR124-3

SFTPC

DLD

WTAP

COL6A5

CAMK2N1

TAB3

HAVCR2

EGR3

MYH10

CLEC4A

CTTN

TBXAS1

ADAMTS8

CA2

AGFG1

SEMA7A

STEEP1

TERF2IP

APIP

PPID

HSP90AB1

GAB2

OGDH

MTF1

BMPER

CHRM2

RNF182

NDUFS4

BGN

SLC8A3

FCN2

APRT

S1PR3

CARTPT

NPR2

C1D

SLC6A2

FOXN2

STMN1

FLRT3

DDT

CACNA1B

MIR488

MIR196A2

XPNPEP2

GRIA1

EEF1A1

RYK

BCKDHB

ROR1

GORASP1

IRAK4

PCYT1A

IL1F10

IMMT

GABBR2

CMKLR1

ATG3

ATG5

APOO

PIM1

MIR494

APOC1

MICU1

CANT1

FGF21

HCP5

PRCP

GRIK2

PROS1

CFB

TSPAN18

SRF

MIR1246

EGLN3

CDK5R1

MIR873

SGCB

HSPA9

EGLN1

IGFBP7

CFP

ACSS2

SLC25A3

PPP1CC

AMBP

CGB5

TRPA1

SH3GLB1

CD226

LINC-ROR

NID2

SLC22A8

ARG2

APOL4

SESN2

ADAMTS20

TRIM11

UTS2B

SAP30BP

MIR548B

PRKDC

ADAMTSL1

SIGMAR1

MASP1

CSNK2A2

PRDX1

ZNF354A

HDAC6

PRDX2

SSPOP

AHNAK

MYL4

PSMA7

CHADL

MIR489

GRM5

PARP2

FLNC

KCNK2

MYL3

THBS4

FAIM2

MIR665

MIR544A

COL20A1

SMDT1

RACK1

PPRC1

HAVCR1

UBE2G2

SPRED2

PSMC3

ADAM11

FNDC5

PRR34-AS1

CTRL

DNM2

CAMKK2

PGAM5

GSTT1

KCNA2

FREM3

FCN3

CELSR1

UCP1

SUMO2

AKIP1

P2RY11

FEM1A

ANK2

ZBTB33

GCKR

MAPK7

MIR486-2

UCN2

UMOD

ORAI1

TRIM63

HBG2

GCH1

NLRC5

ATR

GOT1

BIK

SLC12A4

PAFAH2

ANXA7

PDE3B

ENTPD2

MIR181C

GPER1

CHRDL1

PTP4A2

ACOD1

TRPV2

PDE6B

TRPC5

MIR216A

CTH

IL22RA1

ANG

OIP5-AS1

NUMA1

B4GALT6

ADPRS

DBI

SNHG4

MAP1A

LIFR

BPIFB4

MIR137

ANK3

OTUD7B

NSF

JMJD6

MIR133A1HG

ABCB10

TNFSF9

FXYD4

HPX

IGF2BP2

MAP3K20

SUMO4

PSMC5

TFEB

PLD2

RAMP1

HRG

MIR496

MIR30D

CNDP1

SUZ12

PTPRO

A2M

HAX1

MT3

MIR613

ORM1

THUMPD3-AS1

ATP5MC1

MIR134

FIS1

PDGFD

PKP2

MIR124-2

TRIB3

CSF2RB

KCNA5

ATG12

NDRG2

MIR1-2

MIR133A2

RACGAP1

BCL2L13

ATP5F1B

PHB2

FKBP1B

SLC18A2

RBP3

PSMA2

LINC00461

TMBIM6

MAFK

DGKQ

A2M-AS1

CLCF1

PAX2

EPHA4

FAM3A

SAA4

MMP16

RALBP1

ADAMTS1

ENO3

STEAP4

YEATS2

MIR1306

FCN1

FTX

MIR217

TXNL1

TIMP4

CPT1B

PLA2G15

MIR30C2

UBE2D3

MIR208B

MICU2

LTB4R2

SUCNR1

TANK

AASS

GRIA4

TRADD

MIR766

C1QA

NOX3

NPY1R

HEXIM1

KCNRG

HSPA1L

B3GALT4

EXOC5

CADPS

CSN3

HIF3A

ZNF667

H2BC3

RAB38

ST2

ART3

USP18

EIF5

FTO

BLVRA

PNPLA8

SERPINB9P1

ATG4C

IMMP2L

DNAJC7

MIR153-1

EPS15L1

MYL1

ATP5F1A

HSPB8

TRIM72

SLC12A5

CKLF

MIEF1

PARG

ATP2A1

HSD17B14

RRAD

THAP5

HIC2

NOP58

MCUB

MIR320B1

BDNF-AS

LOC111365141

NFE2L1

TLK1

FSTL1

TNNI3K

PXMP2

SYDE1

TNNI1

AGXT2

ENTPD7

MIR199A2

MIR206

FST

MIR1203

PDHA1

DDAH2

MIR1290

JPH2

PADI4

CNRIP1

SCIRT

KCNA3

CD5L

RIMS1

INHBB

MIR125B2

C1QTNF1

MIR638

TOX4

LAMTOR5

NDUFA5

DAO

YTHDF3

PRG4

PAMR1

UBE2O

MIEF2

TPR

STIP1

POU4F2

CHMP2A

LAIR1

MIR885

CAMK2B

HSPA13

ADAMTS5

RGS16

MIR297

CNPY3

CKMT2

ATG9A

KCNA4

MIR320B2

CASP14

USP29

MIR1275

TNNC1

SCML2

LINC00520

MIR1283-1

KCNB2

Supplementary table 2 Result of GO-BP enrichment analysis in DAVID database.

Category	Term
GOTERM_BP_DIRECT	GO:0042493~response to drug
GOTERM_BP_DIRECT	GO:0009410~response to xenobiotic stimulus
GOTERM_BP_DIRECT	GO:0043066~negative regulation of apoptotic process
GOTERM_BP_DIRECT	GO:0010628~positive regulation of gene expression
GOTERM_BP_DIRECT	GO:0008284~positive regulation of cell proliferation
GOTERM_BP_DIRECT	GO:0048661~positive regulation of smooth muscle cell proliferation
GOTERM_BP_DIRECT	GO:0043065~positive regulation of apoptotic process
GOTERM_BP_DIRECT	GO:0001666~response to hypoxia
GOTERM_BP_DIRECT	GO:0010629~negative regulation of gene expression

GOTERM_BP_DIRECT	GO:0030335~positive regulation of cell migration
GOTERM_BP_DIRECT	GO:0006915~apoptotic process
GOTERM_BP_DIRECT	GO:0018105~peptidyl-serine phosphorylation
GOTERM_BP_DIRECT	GO:0018107~peptidyl-threonine phosphorylation
GOTERM_BP_DIRECT	GO:0007568~aging
GOTERM_BP_DIRECT	GO:0001938~positive regulation of endothelial cell proliferation
GOTERM_BP_DIRECT	GO:0043536~positive regulation of blood vessel endothelial cell migration
GOTERM_BP_DIRECT	GO:0001934~positive regulation of protein phosphorylation
GOTERM_BP_DIRECT	GO:0042542~response to hydrogen peroxide
GOTERM_BP_DIRECT	GO:0006508~proteolysis
GOTERM_BP_DIRECT	GO:0006954~inflammatory response

GOTERM_BP_DIRECT	GO:0071456~cellular response to hypoxia
GOTERM_BP_DIRECT	GO:0032355~response to estradiol
GOTERM_BP_DIRECT	GO:0007507~heart development
GOTERM_BP_DIRECT	GO:0001837~epithelial to mesenchymal transition
GOTERM_BP_DIRECT	I902895~positive regulation of pri-miRNA transcription from RNA polymerase II prom
GOTERM_BP_DIRECT	GO:0045429~positive regulation of nitric oxide biosynthetic process
GOTERM_BP_DIRECT	GO:0014068~positive regulation of phosphatidylinositol 3-kinase signaling
GOTERM_BP_DIRECT	GO:0010862~positive regulation of pathway-restricted SMAD protein phosphorylation
GOTERM_BP_DIRECT	GO:0046777~protein autophosphorylation
GOTERM_BP_DIRECT	GO:0071260~cellular response to mechanical stimulus
GOTERM_BP_DIRECT	GO:0033138~positive regulation of peptidyl-serine phosphorylation

GOTERM_BP_DIRECT	GO:0030307~positive regulation of cell growth
GOTERM_BP_DIRECT	GO:0006468~protein phosphorylation
GOTERM_BP_DIRECT	GO:0032496~response to lipopolysaccharide
GOTERM_BP_DIRECT	GO:0045893~positive regulation of transcription, DNA-templated
GOTERM_BP_DIRECT	GO:0030574~collagen catabolic process
GOTERM_BP_DIRECT	GO:0033280~response to vitamin D
GOTERM_BP_DIRECT	GO:0060391~positive regulation of SMAD protein import into nucleus
GOTERM_BP_DIRECT	GO:0051897~positive regulation of protein kinase B signaling
GOTERM_BP_DIRECT	GO:0001525~angiogenesis
GOTERM_BP_DIRECT	GO:0007173~epidermal growth factor receptor signaling pathway
GOTERM_BP_DIRECT	GO:0007165~signal transduction

GOTERM_BP_DIRECT	GO:0030509~BMP signaling pathway
GOTERM_BP_DIRECT	GO:0010718~positive regulation of epithelial to mesenchymal transition
GOTERM_BP_DIRECT	GO:0033591~response to L-ascorbic acid
GOTERM_BP_DIRECT	GO:0043280~positive regulation of cysteine-type endopeptidase activity involved in apoptotic process
GOTERM_BP_DIRECT	GO:0043524~negative regulation of neuron apoptotic process
GOTERM_BP_DIRECT	GO:0008285~negative regulation of cell proliferation
GOTERM_BP_DIRECT	GO:1901522~positive regulation of transcription from RNA polymerase II promoter involved in cellular response to chemical stimulus
GOTERM_BP_DIRECT	GO:0032148~activation of protein kinase B activity
GOTERM_BP_DIRECT	GO:0009612~response to mechanical stimulus
GOTERM_BP_DIRECT	GO:0030163~protein catabolic process
GOTERM_BP_DIRECT	GO:0035924~cellular response to vascular endothelial growth factor stimulus

GOTERM_BP_DIRECT	GO:0043627~response to estrogen
GOTERM_BP_DIRECT	GO:0051402~neuron apoptotic process
GOTERM_BP_DIRECT	GO:0001649~osteoblast differentiation
GOTERM_BP_DIRECT	GO:0045944~positive regulation of transcription from RNA polymerase II promoter
GOTERM_BP_DIRECT	GO:2001237~negative regulation of extrinsic apoptotic signaling pathway
GOTERM_BP_DIRECT	GO:0071549~cellular response to dexamethasone stimulus
GOTERM_BP_DIRECT	GO:0008217~regulation of blood pressure
GOTERM_BP_DIRECT	GO:1902176~negative regulation of oxidative stress-induced intrinsic apoptotic signaling pathway
GOTERM_BP_DIRECT	GO:0045471~response to ethanol
GOTERM_BP_DIRECT	GO:0071392~cellular response to estradiol stimulus
GOTERM_BP_DIRECT	GO:0071498~cellular response to fluid shear stress

GOTERM_BP_DIRECT	GO:0060389~pathway-restricted SMAD protein phosphorylation
GOTERM_BP_DIRECT	GO:0001503~ossification
GOTERM_BP_DIRECT	GO:0007566~embryo implantation
GOTERM_BP_DIRECT	GO:0001541~ovarian follicle development
GOTERM_BP_DIRECT	GO:0051146~striated muscle cell differentiation
GOTERM_BP_DIRECT	GO:0090336~positive regulation of brown fat cell differentiation
GOTERM_BP_DIRECT	GO:0022617~extracellular matrix disassembly
GOTERM_BP_DIRECT	GO:0030512~negative regulation of transforming growth factor beta receptor signaling pathway
GOTERM_BP_DIRECT	GO:0070374~positive regulation of ERK1 and ERK2 cascade
GOTERM_BP_DIRECT	GO:0043410~positive regulation of MAPK cascade
GOTERM_BP_DIRECT	GO:0045737~positive regulation of cyclin-dependent protein serine/threonine kinas[illegible]

	activity
GOTERM_BP_DIRECT	GO:0050714~positive regulation of protein secretion
GOTERM_BP_DIRECT	GO:0007179~transforming growth factor beta receptor signaling pathway
GOTERM_BP_DIRECT	GO:0009408~response to heat
GOTERM_BP_DIRECT	GO:0002062~chondrocyte differentiation
GOTERM_BP_DIRECT	GO:0045766~positive regulation of angiogenesis
GOTERM_BP_DIRECT	GO:0042060~wound healing
GOTERM_BP_DIRECT	GO:0042981~regulation of apoptotic process
GOTERM_BP_DIRECT	GO:0031667~response to nutrient levels
GOTERM_BP_DIRECT	GO:0048146~positive regulation of fibroblast proliferation
GOTERM_BP_DIRECT	GO:0045786~negative regulation of cell cycle
GOTERM_BP_DIRECT	GO:0031929~TOR signaling

GOTERM_BP_DIRECT	GO:0045780~positive regulation of bone resorption
GOTERM_BP_DIRECT	GO:0038083~peptidyl-tyrosine autophosphorylation
GOTERM_BP_DIRECT	GO:0032869~cellular response to insulin stimulus
GOTERM_BP_DIRECT	GO:0014823~response to activity
GOTERM_BP_DIRECT	GO:0045599~negative regulation of fat cell differentiation
GOTERM_BP_DIRECT	GO:0042475~odontogenesis of dentin-containing tooth
GOTERM_BP_DIRECT	GO:0097194~execution phase of apoptosis
GOTERM_BP_DIRECT	GO:0035902~response to immobilization stress
GOTERM_BP_DIRECT	GO:0060395~SMAD protein signal transduction
GOTERM_BP_DIRECT	GO:0051384~response to glucocorticoid
GOTERM_BP_DIRECT	GO:0043154~negative regulation of cysteine-type endopeptidase activity involved i

	apoptotic process
GOTERM_BP_DIRECT	GO:0048873~homeostasis of number of cells within a tissue
GOTERM_BP_DIRECT	GO:2001243~negative regulation of intrinsic apoptotic signaling pathway
GOTERM_BP_DIRECT	GO:0006469~negative regulation of protein kinase activity
GOTERM_BP_DIRECT	GO:0050679~positive regulation of epithelial cell proliferation
GOTERM_BP_DIRECT	GO:0045892~negative regulation of transcription, DNA-templated
GOTERM_BP_DIRECT	GO:0010575~positive regulation of vascular endothelial growth factor production
GOTERM_BP_DIRECT	GO:0043200~response to amino acid
GOTERM_BP_DIRECT	GO:0035987~endodermal cell differentiation
GOTERM_BP_DIRECT	GO:0051881~regulation of mitochondrial membrane potential
GOTERM_BP_DIRECT	GO:0051281~positive regulation of release of sequestered calcium ion into cytosol
GOTERM_BP_DIRECT	GO:0007584~response to nutrient

GOTERM_BP_DIRECT	GO:0097190~apoptotic signaling pathway
GOTERM_BP_DIRECT	GO:0009749~response to glucose
GOTERM_BP_DIRECT	GO:0045669~positive regulation of osteoblast differentiation
GOTERM_BP_DIRECT	GO:0048511~rhythmic process
GOTERM_BP_DIRECT	GO:0007183~SMAD protein complex assembly
GOTERM_BP_DIRECT	GO:1900182~positive regulation of protein localization to nucleus
GOTERM_BP_DIRECT	GO:0071773~cellular response to BMP stimulus
GOTERM_BP_DIRECT	GO:0043388~positive regulation of DNA binding
GOTERM_BP_DIRECT	GO:0043406~positive regulation of MAP kinase activity
GOTERM_BP_DIRECT	GO:0071356~cellular response to tumor necrosis factor
GOTERM_BP_DIRECT	GO:0048762~mesenchymal cell differentiation

GOTERM_BP_DIRECT	GO:0002674~negative regulation of acute inflammatory response
GOTERM_BP_DIRECT	GO:0048662~negative regulation of smooth muscle cell proliferation
GOTERM_BP_DIRECT	GO:0001657~ureteric bud development
GOTERM_BP_DIRECT	GO:0006730~one-carbon metabolic process
GOTERM_BP_DIRECT	GO:0032868~response to insulin
GOTERM_BP_DIRECT	GO:0060412~ventricular septum morphogenesis
GOTERM_BP_DIRECT	GO:0098586~cellular response to virus
GOTERM_BP_DIRECT	GO:0051726~regulation of cell cycle
GOTERM_BP_DIRECT	GO:0034614~cellular response to reactive oxygen species
GOTERM_BP_DIRECT	GO:0001542~ovulation from ovarian follicle
GOTERM_BP_DIRECT	GO:0070168~negative regulation of biomineral tissue development

GOTERM_BP_DIRECT	GO:0008625~extrinsic apoptotic signaling pathway via death domain receptors
GOTERM_BP_DIRECT	GO:0001890~placenta development
GOTERM_BP_DIRECT	GO:0031663~lipopolysaccharide-mediated signaling pathway
GOTERM_BP_DIRECT	GO:0035094~response to nicotine
GOTERM_BP_DIRECT	GO:0042311~vasodilation
GOTERM_BP_DIRECT	GO:0071276~cellular response to cadmium ion
GOTERM_BP_DIRECT	GO:0050731~positive regulation of peptidyl-tyrosine phosphorylation
GOTERM_BP_DIRECT	GO:0030501~positive regulation of bone mineralization
GOTERM_BP_DIRECT	GO:0007281~germ cell development
GOTERM_BP_DIRECT	GO:0035556~intracellular signal transduction
GOTERM_BP_DIRECT	GO:0042307~positive regulation of protein import into nucleus

GOTERM_BP_DIRECT	GO:0070723~response to cholesterol
GOTERM_BP_DIRECT	GO:0033197~response to vitamin E
GOTERM_BP_DIRECT	GO:0009411~response to UV
GOTERM_BP_DIRECT	GO:0043407~negative regulation of MAP kinase activity
GOTERM_BP_DIRECT	GO:1904706~negative regulation of vascular smooth muscle cell proliferation
GOTERM_BP_DIRECT	GO:0031100~animal organ regeneration
GOTERM_BP_DIRECT	GO:0003181~atrioventricular valve morphogenesis
GOTERM_BP_DIRECT	GO:0032495~response to muramyl dipeptide
GOTERM_BP_DIRECT	GO:0071364~cellular response to epidermal growth factor stimulus
GOTERM_BP_DIRECT	GO:0050727~regulation of inflammatory response
GOTERM_BP_DIRECT	GO:0050673~epithelial cell proliferation

GOTERM_BP_DIRECT	GO:0008630~intrinsic apoptotic signaling pathway in response to DNA damage
GOTERM_BP_DIRECT	GO:0048011~neurotrophin TRK receptor signaling pathway
GOTERM_BP_DIRECT	GO:0048546~digestive tract morphogenesis
GOTERM_BP_DIRECT	GO:0097191~extrinsic apoptotic signaling pathway
GOTERM_BP_DIRECT	GO:0043491~protein kinase B signaling
GOTERM_BP_DIRECT	GO:0060317~cardiac epithelial to mesenchymal transition
GOTERM_BP_DIRECT	GO:0071222~cellular response to lipopolysaccharide
GOTERM_BP_DIRECT	GO:0030282~bone mineralization
GOTERM_BP_DIRECT	GO:0090398~cellular senescence
GOTERM_BP_DIRECT	GO:0007219~Notch signaling pathway
GOTERM_BP_DIRECT	GO:0071407~cellular response to organic cyclic compound

GOTERM_BP_DIRECT	GO:1900034~regulation of cellular response to heat
GOTERM_BP_DIRECT	GO:0032270~positive regulation of cellular protein metabolic process
GOTERM_BP_DIRECT	GO:1904645~response to beta-amyloid
GOTERM_BP_DIRECT	GO:0001822~kidney development
GOTERM_BP_DIRECT	GO:0045165~cell fate commitment
GOTERM_BP_DIRECT	GO:1903078~positive regulation of protein localization to plasma membrane
GOTERM_BP_DIRECT	GO:0071363~cellular response to growth factor stimulus
GOTERM_BP_DIRECT	GO:1902166~negative regulation of intrinsic apoptotic signaling pathway in respons to DNA damage by p53 class mediator
GOTERM_BP_DIRECT	GO:0000302~response to reactive oxygen species
GOTERM_BP_DIRECT	GO:1904019~epithelial cell apoptotic process
GOTERM_BP_DIRECT	GO:0008631~intrinsic apoptotic signaling pathway in response to oxidative stress

GOTERM_BP_DIRECT	GO:0051216~cartilage development
GOTERM_BP_DIRECT	GO:0050728~negative regulation of inflammatory response
GOTERM_BP_DIRECT	GO:0003203~endocardial cushion morphogenesis
GOTERM_BP_DIRECT	GO:0048545~response to steroid hormone
GOTERM_BP_DIRECT	GO:0035162~embryonic hemopoiesis
GOTERM_BP_DIRECT	GO:0030168~platelet activation
GOTERM_BP_DIRECT	GO:1901215~negative regulation of neuron death
GOTERM_BP_DIRECT	GO:0007169~transmembrane receptor protein tyrosine kinase signaling pathway
GOTERM_BP_DIRECT	GO:0048812~neuron projection morphogenesis
GOTERM_BP_DIRECT	GO:2000811~negative regulation of anoikis
GOTERM_BP_DIRECT	GO:0042752~regulation of circadian rhythm

GOTERM_BP_DIRECT	GO:0001501~skeletal system development
GOTERM_BP_DIRECT	GO:0055093~response to hyperoxia
GOTERM_BP_DIRECT	GO:0009611~response to wounding
GOTERM_BP_DIRECT	GO:1902894~negative regulation of pri-miRNA transcription from RNA polymerase promoter
GOTERM_BP_DIRECT	GO:0032930~positive regulation of superoxide anion generation
GOTERM_BP_DIRECT	GO:0009791~post-embryonic development
GOTERM_BP_DIRECT	GO:0045216~cell-cell junction organization
GOTERM_BP_DIRECT	GO:0001836~release of cytochrome c from mitochondria
GOTERM_BP_DIRECT	GO:0030279~negative regulation of ossification
GOTERM_BP_DIRECT	GO:0045861~negative regulation of proteolysis
GOTERM_BP_DIRECT	GO:0051247~positive regulation of protein metabolic process

GOTERM_BP_DIRECT	GO:0042554~superoxide anion generation
GOTERM_BP_DIRECT	GO:0014911~positive regulation of smooth muscle cell migration
GOTERM_BP_DIRECT	GO:0007569~cell aging
GOTERM_BP_DIRECT	GO:0042026~protein refolding
GOTERM_BP_DIRECT	GO:0001933~negative regulation of protein phosphorylation
GOTERM_BP_DIRECT	GO:0051092~positive regulation of NF-kappaB transcription factor activity
GOTERM_BP_DIRECT	GO:0016485~protein processing
GOTERM_BP_DIRECT	GO:0051000~positive regulation of nitric-oxide synthase activity
GOTERM_BP_DIRECT	GO:0001782~B cell homeostasis
GOTERM_BP_DIRECT	GO:0046579~positive regulation of Ras protein signal transduction
GOTERM_BP_DIRECT	GO:0042127~regulation of cell proliferation

GOTERM_BP_DIRECT	GO:0032880~regulation of protein localization
GOTERM_BP_DIRECT	GO:0010875~positive regulation of cholesterol efflux
GOTERM_BP_DIRECT	GO:0030198~extracellular matrix organization
GOTERM_BP_DIRECT	GO:0046686~response to cadmium ion
GOTERM_BP_DIRECT	GO:0090200~positive regulation of release of cytochrome c from mitochondria
GOTERM_BP_DIRECT	GO:0009636~response to toxic substance
GOTERM_BP_DIRECT	GO:0016477~cell migration
GOTERM_BP_DIRECT	GO:1903672~positive regulation of sprouting angiogenesis
GOTERM_BP_DIRECT	GO:0046697~decidualization
GOTERM_BP_DIRECT	GO:0010332~response to gamma radiation
GOTERM_BP_DIRECT	GO:0070372~regulation of ERK1 and ERK2 cascade

GOTERM_BP_DIRECT	GO:0048041~focal adhesion assembly
GOTERM_BP_DIRECT	GO:0030182~neuron differentiation
GOTERM_BP_DIRECT	GO:0001958~endochondral ossification
GOTERM_BP_DIRECT	GO:0050900~leukocyte migration
GOTERM_BP_DIRECT	GO:0048010~vascular endothelial growth factor receptor signaling pathway
GOTERM_BP_DIRECT	GO:0051602~response to electrical stimulus
GOTERM_BP_DIRECT	GO:0010592~positive regulation of lamellipodium assembly
GOTERM_BP_DIRECT	GO:0032088~negative regulation of NF-kappaB transcription factor activity
GOTERM_BP_DIRECT	GO:0001889~liver development
GOTERM_BP_DIRECT	GO:0006974~cellular response to DNA damage stimulus
GOTERM_BP_DIRECT	GO:0048701~embryonic cranial skeleton morphogenesis

GOTERM_BP_DIRECT	GO:0043278~response to morphine
GOTERM_BP_DIRECT	GO:0042327~positive regulation of phosphorylation
GOTERM_BP_DIRECT	GO:0051968~positive regulation of synaptic transmission, glutamatergic
GOTERM_BP_DIRECT	GO:0035633~maintenance of permeability of blood-brain barrier
GOTERM_BP_DIRECT	GO:1904193~negative regulation of cholangiocyte apoptotic process
GOTERM_BP_DIRECT	GO:0046370~fructose biosynthetic process
GOTERM_BP_DIRECT	GO:0006808~regulation of nitrogen utilization
GOTERM_BP_DIRECT	GO:0043101~purine-containing compound salvage
GOTERM_BP_DIRECT	GO:0071000~response to magnetism
GOTERM_BP_DIRECT	GO:0045666~positive regulation of neuron differentiation
GOTERM_BP_DIRECT	GO:0003180~aortic valve morphogenesis

GOTERM_BP_DIRECT	GO:0016239~positive regulation of macroautophagy
GOTERM_BP_DIRECT	GO:0043029~T cell homeostasis
GOTERM_BP_DIRECT	GO:0032007~negative regulation of TOR signaling
GOTERM_BP_DIRECT	GO:2001234~negative regulation of apoptotic signaling pathway
GOTERM_BP_DIRECT	GO:0060045~positive regulation of cardiac muscle cell proliferation
GOTERM_BP_DIRECT	GO:0048147~negative regulation of fibroblast proliferation
GOTERM_BP_DIRECT	GO:0097192~extrinsic apoptotic signaling pathway in absence of ligand
GOTERM_BP_DIRECT	GO:2000379~positive regulation of reactive oxygen species metabolic process
GOTERM_BP_DIRECT	GO:0030010~establishment of cell polarity
GOTERM_BP_DIRECT	GO:0034612~response to tumor necrosis factor
GOTERM_BP_DIRECT	GO:0048469~cell maturation

GOTERM_BP_DIRECT	GO:0030154~cell differentiation
GOTERM_BP_DIRECT	GO:0000122~negative regulation of transcription from RNA polymerase II promoter
GOTERM_BP_DIRECT	GO:0032570~response to progesterone
GOTERM_BP_DIRECT	GO:0046427~positive regulation of JAK-STAT cascade
GOTERM_BP_DIRECT	GO:0045907~positive regulation of vasoconstriction
GOTERM_BP_DIRECT	GO:0046326~positive regulation of glucose import
GOTERM_BP_DIRECT	GO:0033077~T cell differentiation in thymus
GOTERM_BP_DIRECT	GO:0045069~regulation of viral genome replication
GOTERM_BP_DIRECT	GO:0071887~leukocyte apoptotic process
GOTERM_BP_DIRECT	GO:0070141~response to UV-A
GOTERM_BP_DIRECT	GO:0006157~deoxyadenosine catabolic process

GOTERM_BP_DIRECT	GO:0021502~neural fold elevation formation
GOTERM_BP_DIRECT	GO:0002051~osteoblast fate commitment
GOTERM_BP_DIRECT	GO:0046666~retinal cell programmed cell death
GOTERM_BP_DIRECT	GO:1902263~apoptotic process involved in embryonic digit morphogenesis
GOTERM_BP_DIRECT	GO:0048286~lung alveolus development
GOTERM_BP_DIRECT	GO:0010634~positive regulation of epithelial cell migration
GOTERM_BP_DIRECT	GO:0008584~male gonad development
GOTERM_BP_DIRECT	GO:1901796~regulation of signal transduction by p53 class mediator
GOTERM_BP_DIRECT	GO:0071479~cellular response to ionizing radiation
GOTERM_BP_DIRECT	GO:0042593~glucose homeostasis
GOTERM_BP_DIRECT	GO:1901223~negative regulation of NIK/NF-kappaB signaling

GOTERM_BP_DIRECT	GO:0001701~in utero embryonic development
GOTERM_BP_DIRECT	GO:0009409~response to cold
GOTERM_BP_DIRECT	GO:1901216~positive regulation of neuron death
GOTERM_BP_DIRECT	GO:0051091~positive regulation of sequence-specific DNA binding transcription factor activity
GOTERM_BP_DIRECT	GO:0007275~multicellular organism development
GOTERM_BP_DIRECT	GO:0048863~stem cell differentiation
GOTERM_BP_DIRECT	GO:0006979~response to oxidative stress
GOTERM_BP_DIRECT	GO:0045747~positive regulation of Notch signaling pathway
GOTERM_BP_DIRECT	GO:0009725~response to hormone
GOTERM_BP_DIRECT	GO:0048597~post-embryonic camera-type eye morphogenesis
GOTERM_BP_DIRECT	GO:0033590~response to cobalamin

GOTERM_BP_DIRECT	GO:0071228~cellular response to tumor cell
GOTERM_BP_DIRECT	GO:0046059~dAMP catabolic process
GOTERM_BP_DIRECT	GO:0003157~endocardium development
GOTERM_BP_DIRECT	GO:0042482~positive regulation of odontogenesis
GOTERM_BP_DIRECT	GO:0002906~negative regulation of mature B cell apoptotic process
GOTERM_BP_DIRECT	GO:2001225~regulation of chloride transport
GOTERM_BP_DIRECT	GO:0090400~stress-induced premature senescence
GOTERM_BP_DIRECT	GO:0060485~mesenchyme development
GOTERM_BP_DIRECT	GO:0030890~positive regulation of B cell proliferation
GOTERM_BP_DIRECT	GO:0045087~innate immune response
GOTERM_BP_DIRECT	GO:0051591~response to cAMP

GOTERM_BP_DIRECT	GO:0006749~glutathione metabolic process
GOTERM_BP_DIRECT	GO:0030308~negative regulation of cell growth
GOTERM_BP_DIRECT	GO:0048538~thymus development
GOTERM_BP_DIRECT	GO:0003151~outflow tract morphogenesis
GOTERM_BP_DIRECT	GO:0048013~ephrin receptor signaling pathway
GOTERM_BP_DIRECT	GO:1900087~positive regulation of G1/S transition of mitotic cell cycle
GOTERM_BP_DIRECT	GO:0036305~ameloblast differentiation
GOTERM_BP_DIRECT	GO:0097411~hypoxia-inducible factor-1alpha signaling pathway
GOTERM_BP_DIRECT	GO:0060687~regulation of branching involved in prostate gland morphogenesis
GOTERM_BP_DIRECT	GO:1905605~positive regulation of maintenance of permeability of blood-brain barrier
GOTERM_BP_DIRECT	GO:0042743~hydrogen peroxide metabolic process

GOTERM_BP_DIRECT	GO:1901984~negative regulation of protein acetylation
GOTERM_BP_DIRECT	GO:0032263~GMP salvage
GOTERM_BP_DIRECT	GO:0090335~regulation of brown fat cell differentiation
GOTERM_BP_DIRECT	GO:0014042~positive regulation of neuron maturation
GOTERM_BP_DIRECT	GO:0002248~connective tissue replacement involved in inflammatory response wound healing
GOTERM_BP_DIRECT	GO:0044209~AMP salvage
GOTERM_BP_DIRECT	GO:0072734~cellular response to staurosporine
GOTERM_BP_DIRECT	GO:1905007~positive regulation of epithelial to mesenchymal transition involved in endocardial cushion formation
GOTERM_BP_DIRECT	GO:0072089~stem cell proliferation
GOTERM_BP_DIRECT	GO:0031648~protein destabilization
GOTERM_BP_DIRECT	GO:0043124~negative regulation of I-kappaB kinase/NF-kappaB signaling

GOTERM_BP_DIRECT	GO:0006366~transcription from RNA polymerase II promoter
GOTERM_BP_DIRECT	GO:0010507~negative regulation of autophagy
GOTERM_BP_DIRECT	GO:0031334~positive regulation of protein complex assembly
GOTERM_BP_DIRECT	GO:0060348~bone development
GOTERM_BP_DIRECT	GO:1904707~positive regulation of vascular smooth muscle cell proliferation
GOTERM_BP_DIRECT	GO:0045600~positive regulation of fat cell differentiation
GOTERM_BP_DIRECT	GO:0061419~positive regulation of transcription from RNA polymerase II promoter i response to hypoxia
GOTERM_BP_DIRECT	GO:0007231~osmosensory signaling pathway
GOTERM_BP_DIRECT	GO:0071393~cellular response to progesterone stimulus
GOTERM_BP_DIRECT	GO:0051902~negative regulation of mitochondrial depolarization
GOTERM_BP_DIRECT	GO:0014031~mesenchymal cell development

GOTERM_BP_DIRECT	GO:0090090~negative regulation of canonical Wnt signaling pathway
GOTERM_BP_DIRECT	GO:0071230~cellular response to amino acid stimulus
GOTERM_BP_DIRECT	GO:0022900~electron transport chain
GOTERM_BP_DIRECT	GO:0030216~keratinocyte differentiation
GOTERM_BP_DIRECT	GO:0008286~insulin receptor signaling pathway
GOTERM_BP_DIRECT	GO:0007182~common-partner SMAD protein phosphorylation
GOTERM_BP_DIRECT	GO:0042487~regulation of odontogenesis of dentin-containing tooth
GOTERM_BP_DIRECT	GO:0010742~macrophage derived foam cell differentiation
GOTERM_BP_DIRECT	GO:0003344~pericardium morphogenesis
GOTERM_BP_DIRECT	GO:0003179~heart valve morphogenesis
GOTERM_BP_DIRECT	GO:1990776~response to angiotensin

GOTERM_BP_DIRECT	GO:0007435~salivary gland morphogenesis
GOTERM_BP_DIRECT	GO:0071455~cellular response to hyperoxia
GOTERM_BP_DIRECT	GO:0071902~positive regulation of protein serine/threonine kinase activity
GOTERM_BP_DIRECT	GO:0001570~vasculogenesis
GOTERM_BP_DIRECT	GO:0009414~response to water deprivation
GOTERM_BP_DIRECT	GO:0060336~negative regulation of interferon-gamma-mediated signaling pathway
GOTERM_BP_DIRECT	GO:0060965~negative regulation of gene silencing by miRNA
GOTERM_BP_DIRECT	GO:0061684~chaperone-mediated autophagy
GOTERM_BP_DIRECT	GO:0051152~positive regulation of smooth muscle cell differentiation
GOTERM_BP_DIRECT	GO:0006166~purine ribonucleoside salvage
GOTERM_BP_DIRECT	GO:0032873~negative regulation of stress-activated MAPK cascade

GOTERM_BP_DIRECT	GO:0060740~prostate gland epithelium morphogenesis
GOTERM_BP_DIRECT	GO:0045839~negative regulation of mitotic nuclear division
GOTERM_BP_DIRECT	GO:1904179~positive regulation of adipose tissue development
GOTERM_BP_DIRECT	GO:0000255~allantoin metabolic process
GOTERM_BP_DIRECT	GO:0010544~negative regulation of platelet activation
GOTERM_BP_DIRECT	GO:0032364~oxygen homeostasis
GOTERM_BP_DIRECT	GO:0061384~heart trabecula morphogenesis
GOTERM_BP_DIRECT	GO:0030858~positive regulation of epithelial cell differentiation
GOTERM_BP_DIRECT	GO:0022898~regulation of transmembrane transporter activity
GOTERM_BP_DIRECT	GO:0007611~learning or memory
GOTERM_BP_DIRECT	GO:0006006~glucose metabolic process

GOTERM_BP_DIRECT	GO:0001947~heart looping
GOTERM_BP_DIRECT	GO:0071560~cellular response to transforming growth factor beta stimulus
GOTERM_BP_DIRECT	GO:1901224~positive regulation of NIK/NF-kappaB signaling
GOTERM_BP_DIRECT	GO:0008354~germ cell migration
GOTERM_BP_DIRECT	GO:0060982~coronary artery morphogenesis
GOTERM_BP_DIRECT	GO:0046903~secretion
GOTERM_BP_DIRECT	GO:0051148~negative regulation of muscle cell differentiation
GOTERM_BP_DIRECT	GO:0002070~epithelial cell maturation
GOTERM_BP_DIRECT	GO:0010224~response to UV-B
GOTERM_BP_DIRECT	GO:0034405~response to fluid shear stress
GOTERM_BP_DIRECT	GO:0003208~cardiac ventricle morphogenesis

GOTERM_BP_DIRECT	GO:0010042~response to manganese ion
GOTERM_BP_DIRECT	GO:0021766~hippocampus development
GOTERM_BP_DIRECT	GO:0006457~protein folding
GOTERM_BP_DIRECT	GO:0033674~positive regulation of kinase activity
GOTERM_BP_DIRECT	GO:0000187~activation of MAPK activity
GOTERM_BP_DIRECT	GO:0070301~cellular response to hydrogen peroxide
GOTERM_BP_DIRECT	GO:0045792~negative regulation of cell size
GOTERM_BP_DIRECT	GO:2000048~negative regulation of cell-cell adhesion mediated by cadherin
GOTERM_BP_DIRECT	GO:0046638~positive regulation of alpha-beta T cell differentiation
GOTERM_BP_DIRECT	GO:0044346~fibroblast apoptotic process
GOTERM_BP_DIRECT	GO:0031000~response to caffeine

GOTERM_BP_DIRECT	GO:0014074~response to purine-containing compound
GOTERM_BP_DIRECT	GO:0003214~cardiac left ventricle morphogenesis
GOTERM_BP_DIRECT	GO:0051272~positive regulation of cellular component movement
GOTERM_BP_DIRECT	GO:0033033~negative regulation of myeloid cell apoptotic process
GOTERM_BP_DIRECT	GO:0060068~vagina development
GOTERM_BP_DIRECT	GO:0070244~negative regulation of thymocyte apoptotic process
GOTERM_BP_DIRECT	GO:0002467~germinal center formation
GOTERM_BP_DIRECT	GO:0042118~endothelial cell activation
GOTERM_BP_DIRECT	GO:0003272~endocardial cushion formation
GOTERM_BP_DIRECT	GO:0031666~positive regulation of lipopolysaccharide-mediated signaling pathway
GOTERM_BP_DIRECT	GO:0001783~B cell apoptotic process

GOTERM_BP_DIRECT	GO:0050665~hydrogen peroxide biosynthetic process
GOTERM_BP_DIRECT	GO:0010907~positive regulation of glucose metabolic process
GOTERM_BP_DIRECT	GO:0032091~negative regulation of protein binding
GOTERM_BP_DIRECT	GO:0009267~cellular response to starvation
GOTERM_BP_DIRECT	GO:0032092~positive regulation of protein binding
GOTERM_BP_DIRECT	GO:0098869~cellular oxidant detoxification
GOTERM_BP_DIRECT	GO:0071492~cellular response to UV-A
GOTERM_BP_DIRECT	GO:0001823~mesonephros development
GOTERM_BP_DIRECT	GO:0010887~negative regulation of cholesterol storage
GOTERM_BP_DIRECT	GO:1905461~positive regulation of vascular associated smooth muscle cell apoptotic process
GOTERM_BP_DIRECT	GO:1903715~regulation of aerobic respiration

GOTERM_BP_DIRECT	GO:0070242~thymocyte apoptotic process
GOTERM_BP_DIRECT	GO:0072091~regulation of stem cell proliferation
GOTERM_BP_DIRECT	GO:0030518~intracellular steroid hormone receptor signaling pathway
GOTERM_BP_DIRECT	GO:0009651~response to salt stress
GOTERM_BP_DIRECT	GO:1903799~negative regulation of production of miRNAs involved in gene silencin by miRNA
GOTERM_BP_DIRECT	GO:0071223~cellular response to lipoteichoic acid
GOTERM_BP_DIRECT	GO:0042592~homeostatic process
GOTERM_BP_DIRECT	GO:0007166~cell surface receptor signaling pathway
GOTERM_BP_DIRECT	GO:0006952~defense response
GOTERM_BP_DIRECT	GO:0008283~cell proliferation
GOTERM_BP_DIRECT	GO:0030212~hyaluronan metabolic process

GOTERM_BP_DIRECT	GO:0055008~cardiac muscle tissue morphogenesis
GOTERM_BP_DIRECT	GO:0071394~cellular response to testosterone stimulus
GOTERM_BP_DIRECT	GO:1903800~positive regulation of production of miRNAs involved in gene silencin[illegible] by miRNA
GOTERM_BP_DIRECT	GO:0043117~positive regulation of vascular permeability
GOTERM_BP_DIRECT	GO:0048711~positive regulation of astrocyte differentiation
GOTERM_BP_DIRECT	GO:0051549~positive regulation of keratinocyte migration
GOTERM_BP_DIRECT	GO:1990314~cellular response to insulin-like growth factor stimulus
GOTERM_BP_DIRECT	GO:0005979~regulation of glycogen biosynthetic process
GOTERM_BP_DIRECT	GO:0043276~anoikis
GOTERM_BP_DIRECT	GO:0006919~activation of cysteine-type endopeptidase activity involved in apoptoti[illegible] process
GOTERM_BP_DIRECT	GO:0098609~cell-cell adhesion

GOTERM_BP_DIRECT	GO:0045056~transcytosis
GOTERM_BP_DIRECT	GO:0051974~negative regulation of telomerase activity
GOTERM_BP_DIRECT	GO:0002221~pattern recognition receptor signaling pathway
GOTERM_BP_DIRECT	GO:0002357~defense response to tumor cell
GOTERM_BP_DIRECT	GO:0033690~positive regulation of osteoblast proliferation
GOTERM_BP_DIRECT	GO:0019430~removal of superoxide radicals
GOTERM_BP_DIRECT	GO:1903265~positive regulation of tumor necrosis factor-mediated signaling pathway
GOTERM_BP_DIRECT	GO:0010745~negative regulation of macrophage derived foam cell differentiation
GOTERM_BP_DIRECT	GO:0060411~cardiac septum morphogenesis
GOTERM_BP_DIRECT	GO:0071333~cellular response to glucose stimulus
GOTERM_BP_DIRECT	GO:0000902~cell morphogenesis

GOTERM_BP_DIRECT	GO:0007596~blood coagulation
GOTERM_BP_DIRECT	GO:0051926~negative regulation of calcium ion transport
GOTERM_BP_DIRECT	GO:0003222~ventricular trabecula myocardium morphogenesis
GOTERM_BP_DIRECT	GO:0010035~response to inorganic substance
GOTERM_BP_DIRECT	GO:0003323~type B pancreatic cell development
GOTERM_BP_DIRECT	GO:0097049~motor neuron apoptotic process
GOTERM_BP_DIRECT	GO:0010447~response to acidic pH
GOTERM_BP_DIRECT	GO:0060079~excitatory postsynaptic potential
GOTERM_BP_DIRECT	GO:0071347~cellular response to interleukin-1
GOTERM_BP_DIRECT	GO:0034599~cellular response to oxidative stress
GOTERM_BP_DIRECT	GO:0030324~lung development

GOTERM_BP_DIRECT	GO:0043433~negative regulation of sequence-specific DNA binding transcription factor activity
GOTERM_BP_DIRECT	GO:0045732~positive regulation of protein catabolic process
GOTERM_BP_DIRECT	GO:0010831~positive regulation of myotube differentiation
GOTERM_BP_DIRECT	GO:0016242~negative regulation of macroautophagy
GOTERM_BP_DIRECT	GO:0048708~astrocyte differentiation
GOTERM_BP_DIRECT	GO:0070885~negative regulation of calcineurin-NFAT signaling cascade
GOTERM_BP_DIRECT	GO:0043518~negative regulation of DNA damage response, signal transduction by p53 class mediator
GOTERM_BP_DIRECT	GO:0097435~supramolecular fiber organization
GOTERM_BP_DIRECT	GO:1903827~regulation of cellular protein localization
GOTERM_BP_DIRECT	GO:0048266~behavioral response to pain
GOTERM_BP_DIRECT	GO:0003184~pulmonary valve morphogenesis

GOTERM_BP_DIRECT	GO:0031647~regulation of protein stability
GOTERM_BP_DIRECT	GO:0030855~epithelial cell differentiation
GOTERM_BP_DIRECT	GO:0010763~positive regulation of fibroblast migration
GOTERM_BP_DIRECT	GO:0071361~cellular response to ethanol
GOTERM_BP_DIRECT	GO:0030278~regulation of ossification
GOTERM_BP_DIRECT	GO:0060402~calcium ion transport into cytosol
GOTERM_BP_DIRECT	GO:0032230~positive regulation of synaptic transmission, GABAergic
GOTERM_BP_DIRECT	GO:0050862~positive regulation of T cell receptor signaling pathway
GOTERM_BP_DIRECT	GO:0043129~surfactant homeostasis
GOTERM_BP_DIRECT	GO:0030902~hindbrain development
GOTERM_BP_DIRECT	GO:1904385~cellular response to angiotensin

GOTERM_BP_DIRECT	GO:0019395~fatty acid oxidation
GOTERM_BP_DIRECT	GO:0006355~regulation of transcription, DNA-templated
GOTERM_BP_DIRECT	GO:0071346~cellular response to interferon-gamma
GOTERM_BP_DIRECT	GO:0048599~oocyte development
GOTERM_BP_DIRECT	GO:0032515~negative regulation of phosphoprotein phosphatase activity
GOTERM_BP_DIRECT	GO:0046328~regulation of JNK cascade
GOTERM_BP_DIRECT	GO:0044267~cellular protein metabolic process
GOTERM_BP_DIRECT	GO:0048143~astrocyte activation
GOTERM_BP_DIRECT	GO:0071310~cellular response to organic substance
GOTERM_BP_DIRECT	GO:0050872~white fat cell differentiation
GOTERM_BP_DIRECT	GO:0071380~cellular response to prostaglandin E stimulus

GOTERM_BP_DIRECT	GO:0061036~positive regulation of cartilage development
GOTERM_BP_DIRECT	GO:0097067~cellular response to thyroid hormone stimulus
GOTERM_BP_DIRECT	GO:0048009~insulin-like growth factor receptor signaling pathway
GOTERM_BP_DIRECT	GO:0045730~respiratory burst
GOTERM_BP_DIRECT	GO:0060065~uterus development
GOTERM_BP_DIRECT	GO:0043116~negative regulation of vascular permeability
GOTERM_BP_DIRECT	GO:0008637~apoptotic mitochondrial changes
GOTERM_BP_DIRECT	GO:0006805~xenobiotic metabolic process
GOTERM_BP_DIRECT	GO:0031396~regulation of protein ubiquitination
GOTERM_BP_DIRECT	GO:0032469~endoplasmic reticulum calcium ion homeostasis
GOTERM_BP_DIRECT	GO:0045603~positive regulation of endothelial cell differentiation

GOTERM_BP_DIRECT	GO:0048384~retinoic acid receptor signaling pathway
GOTERM_BP_DIRECT	GO:0045987~positive regulation of smooth muscle contraction
GOTERM_BP_DIRECT	GO:0006606~protein import into nucleus
GOTERM_BP_DIRECT	GO:0007049~cell cycle
GOTERM_BP_DIRECT	GO:0002028~regulation of sodium ion transport
GOTERM_BP_DIRECT	GO:0042326~negative regulation of phosphorylation
GOTERM_BP_DIRECT	GO:0031065~positive regulation of histone deacetylation
GOTERM_BP_DIRECT	GO:0043011~myeloid dendritic cell differentiation
GOTERM_BP_DIRECT	GO:0060037~pharyngeal system development
GOTERM_BP_DIRECT	GO:0001516~prostaglandin biosynthetic process
GOTERM_BP_DIRECT	GO:0032332~positive regulation of chondrocyte differentiation

GOTERM_BP_DIRECT	GO:0010039~response to iron ion
GOTERM_BP_DIRECT	GO:0006970~response to osmotic stress
GOTERM_BP_DIRECT	GO:0032760~positive regulation of tumor necrosis factor production
GOTERM_BP_DIRECT	GO:0001892~embryonic placenta development
GOTERM_BP_DIRECT	GO:0033189~response to vitamin A
GOTERM_BP_DIRECT	GO:0035994~response to muscle stretch
GOTERM_BP_DIRECT	GO:0046889~positive regulation of lipid biosynthetic process
GOTERM_BP_DIRECT	GO:0071375~cellular response to peptide hormone stimulus
GOTERM_BP_DIRECT	GO:0090263~positive regulation of canonical Wnt signaling pathway
GOTERM_BP_DIRECT	GO:0050729~positive regulation of inflammatory response
GOTERM_BP_DIRECT	GO:0051607~defense response to virus

GOTERM_BP_DIRECT	GO:0032924~activin receptor signaling pathway
GOTERM_BP_DIRECT	GO:0043401~steroid hormone mediated signaling pathway
GOTERM_BP_DIRECT	GO:1902004~positive regulation of beta-amyloid formation
GOTERM_BP_DIRECT	GO:0006693~prostaglandin metabolic process
GOTERM_BP_DIRECT	GO:0002009~morphogenesis of an epithelium
GOTERM_BP_DIRECT	GO:0002042~cell migration involved in sprouting angiogenesis
GOTERM_BP_DIRECT	GO:0007492~endoderm development
GOTERM_BP_DIRECT	GO:0006809~nitric oxide biosynthetic process
GOTERM_BP_DIRECT	GO:0043254~regulation of protein complex assembly
GOTERM_BP_DIRECT	GO:0031641~regulation of myelination
GOTERM_BP_DIRECT	GO:0002053~positive regulation of mesenchymal cell proliferation

GOTERM_BP_DIRECT	GO:0048754~branching morphogenesis of an epithelial tube
GOTERM_BP_DIRECT	GO:0006801~superoxide metabolic process
GOTERM_BP_DIRECT	GO:0060252~positive regulation of glial cell proliferation

Supplementary table 3 Result of GO-CC enrichment analysis in DAVID database.

Category	Term	Count	%	PValue	Genes
GOTERM_CC_DIRECT	GO:0005829~cytosol	57	64.77273	2.34E-13	GSK3B, NGF, SI HSPA8,
GOTERM_CC_DIRECT	GO:1904813~ficolin-1-rich granule lumen	12	13.63636	4.65E-12	HSPA8,
GOTERM_CC_DIRECT	GO:0005615~extracellular space	32	36.36364	2.38E-11	GSTP1, BMP6, B
GOTERM_CC_DIRECT	GO:0005576~extracellular region	33	37.5	5.08E-11	NOTCH F2, BMP
GOTERM_CC_DIR	GO:0005737~cytoplasm	50	56.818	8.66E-0	GSK3B,

ECT			18	9	TXNIP, F<br>MTOR, T
GOTERM_CC_DIRECT	GO:0005886~plasma membrane	44	50	5.08E-07	GSK3B,<br>CSNK2A<br>NOX1, A
GOTERM_CC_DIRECT	GO:0034774~secretory granule lumen	8	9.090909	5.75E-07	HSPA8,
GOTERM_CC_DIRECT	GO:0032991~macromolecular complex	15	17.04545	1.41E-06	SMAD1,
GOTERM_CC_DIRECT	GO:0043025~neuronal cell body	11	12.5	8.70E-06	HSP90A
GOTERM_CC_DIRECT	GO:0045121~membrane raft	9	10.22727	1.16E-05	ADAM17
GOTERM_CC_DIRECT	GO:0070062~extracellular exosome	24	27.27273	3.44E-05	HSPA8,<br>CTSB
GOTERM_CC_DIRECT	GO:0005764~lysosome	9	10.22727	4.96E-05	HSPA8,
GOTERM_CC_DIRECT	GO:0005634~nucleus	44	50	5.01E-05	GSK3B,<br>HSP90A<br>BCL2, T
GOTERM_CC_DIRECT	GO:0009986~cell surface	12	13.63636	7.87E-05	ADAM17

GOTERM_CC_DIRECT	GO:0031982~vesicle	7	7.954545	9.65E-05	GSTP1,
GOTERM_CC_DIRECT	GO:0016324~apical plasma membrane	9	10.22727	1.93E-04	TJP1, AD
GOTERM_CC_DIRECT	GO:0005739~mitochondrion	17	19.31818	3.11E-04	GSK3B,
GOTERM_CC_DIRECT	GO:0043235~receptor complex	7	7.954545	3.20E-04	NOTCH1
GOTERM_CC_DIRECT	GO:0005901~caveola	5	5.681818	3.20E-04	SRC, NC
GOTERM_CC_DIRECT	GO:0072562~blood microparticle	6	6.818182	3.78E-04	HSPA8,
GOTERM_CC_DIRECT	GO:0030054~cell junction	7	7.954545	4.56E-04	TJP1, OC
GOTERM_CC_DIRECT	GO:0016020~membrane	23	26.13636	7.40E-04	BCHE, H REN, PF
GOTERM_CC_DIRECT	GO:0044294~dendritic growth cone	3	3.409091	7.79E-04	HSP90A
GOTERM_CC_DIRECT	GO:0042470~melanosome	5	5.681818	9.61E-04	HSPA8,
GOTERM_CC_DIRECT	GO:0098978~glutamatergic synapse	8	9.090909	0.001029	MAPK10

GOTERM_CC_DIRECT	GO:0005654~nucleoplasm	30	34.09091	0.001326	GSK3B, NR1H2,
GOTERM_CC_DIRECT	GO:0048471~perinuclear region of cytoplasm	11	12.5	0.001431	HSPA8,
GOTERM_CC_DIRECT	GO:1904724~tertiary granule lumen	4	4.545455	0.001637	MMP8, C
GOTERM_CC_DIRECT	GO:0005788~endoplasmic reticulum lumen	7	7.954545	0.001904	BCHE, A
GOTERM_CC_DIRECT	GO:0030425~dendrite	8	9.090909	0.003362	HSPA8,
GOTERM_CC_DIRECT	GO:0043202~lysosomal lumen	4	4.545455	0.008103	HSPA8,
GOTERM_CC_DIRECT	GO:0005925~focal adhesion	7	7.954545	0.009316	HSPA8,
GOTERM_CC_DIRECT	GO:0005635~nuclear envelope	5	5.681818	0.009469	GPER1,
GOTERM_CC_DIRECT	GO:0043209~myelin sheath	3	3.409091	0.009618	HSP90A
GOTERM_CC_DIRECT	GO:0005796~Golgi lumen	4	4.545455	0.010318	TGFB1,
GOTERM_CC_DIRECT	GO:0070021~transforming growth factor beta1-type II receptor-type I receptor	2	2.272727	0.01264	TGFBR1

	complex				
GOTERM_CC_DIR ECT	GO:0005667~transcription factor complex	5	5.6818 18	0.0169 62	SMAD1,
GOTERM_CC_DIR ECT	GO:0030424~axon	6	6.8181 82	0.0185 78	GSK3B,
GOTERM_CC_DIR ECT	GO:0016323~basolateral plasma membrane	5	5.6818 18	0.0187 01	TJP1, H[illegible]
GOTERM_CC_DIR ECT	GO:1902737~dendritic filopodium	2	2.2727 27	0.0209 79	MAP2, S[illegible]
GOTERM_CC_DIR ECT	GO:0036021~endolysosome lumen	2	2.2727 27	0.0209 79	CTSS, C[illegible]
GOTERM_CC_DIR ECT	GO:0005637~nuclear inner membrane	3	3.4090 91	0.0212 08	SMAD1,
GOTERM_CC_DIR ECT	GO:0043231~intracellular membrane-bounded organelle	10	11.363 64	0.0231 77	BMP2, G[illegible]
GOTERM_CC_DIR ECT	GO:0031012~extracellular matrix	5	5.6818 18	0.0245 66	TGFB1,
GOTERM_CC_DIR ECT	GO:0000791~euchromatin	3	3.4090 91	0.0292 54	HIF1A, E[illegible]
GOTERM_CC_DIR ECT	GO:0072559~NLRP3 inflammasome complex	2	2.2727 27	0.0374 49	CASP1,
GOTERM_CC_DIR	GO:0042995~cell projection	4	4.5454	0.0426	TJP1, P[illegible]

ECT			55	81	
GOTERM_CC_DIRECT	GO:0045177~apical part of cell	3	3.409091	0.043196	TJP1, CA
GOTERM_CC_DIRECT	GO:0009897~external side of plasma membrane	6	6.818182	0.0442	GSR, KD
GOTERM_CC_DIRECT	GO:0005741~mitochondrial outer membrane	4	4.545455	0.048646	RPS6KB
GOTERM_CC_DIRECT	GO:0046930~pore complex	2	2.272727	0.053645	BCL2, BA
GOTERM_CC_DIRECT	GO:0000139~Golgi membrane	7	7.954545	0.057778	ADAM17
GOTERM_CC_DIRECT	GO:0032587~ruffle membrane	3	3.409091	0.065954	ADAM17
GOTERM_CC_DIRECT	GO:0099524~postsynaptic cytosol	2	2.272727	0.069571	HSPA8,
GOTERM_CC_DIRECT	GO:0043020~NADPH oxidase complex	2	2.272727	0.069571	CYBB, N
GOTERM_CC_DIRECT	GO:0043005~neuron projection	5	5.681818	0.076517	MAPK10
GOTERM_CC_DIRECT	GO:0000785~chromatin	9	10.22727	0.079139	SMAD1,
GOTERM_CC_DIR	GO:0016327~apicolateral plasma	2	2.2727	0.0929	TJP1, OC

ECT	membrane		27	65	
GOTERM_CC_DIRECT	GO:0090575~RNA polymerase II transcription factor complex	3	3.409091	0.093087	NR1H2,

Supplementary table 4 Result of GO-MF enrichment analysis in DAVID database.

Category	Term	Count	%	PValue
GOTERM_MF_DIRECT	GO:0019899~enzyme binding	19	21.59091	1.68E-[illegible]
GOTERM_MF_DIRECT	GO:0042802~identical protein binding	32	36.36364	9.06E-[illegible]
GOTERM_MF_DIRECT	GO:0005515~protein binding	80	90.90909	1.13E-[illegible]
GOTERM_MF_DIRECT	GO:0004672~protein kinase activity	13	14.77273	1.66E-[illegible]
GOTERM_MF_DIRECT	GO:0004674~protein serine/threonine kinase activity	13	14.77273	2.38E-[illegible]
GOTERM_MF_DIRECT	GO:0008233~peptidase activity	8	9.090909	4.49E-[illegible]
GOTERM_MF_DIRECT	GO:0005524~ATP binding	23	26.13636	1.34E-[illegible]

GOTERM_MF_DIRECT	GO:0031625~ubiquitin protein ligase binding	11	12.5	1.44E-06
GOTERM_MF_DIRECT	GO:0030235~nitric-oxide synthase regulator activity	4	4.545455	7.80E-06
GOTERM_MF_DIRECT	GO:0004252~serine-type endopeptidase activity	8	9.090909	3.08E-05
GOTERM_MF_DIRECT	GO:0070700~BMP receptor binding	4	4.545455	4.14E-05
GOTERM_MF_DIRECT	GO:0004089~carbonate dehydratase activity	4	4.545455	5.08E-05
GOTERM_MF_DIRECT	GO:0004175~endopeptidase activity	6	6.818182	5.09E-05
GOTERM_MF_DIRECT	GO:0004197~cysteine-type endopeptidase activity	6	6.818182	8.57E-05
GOTERM_MF_DIRECT	GO:0005178~integrin binding	7	7.954545	1.00E-04
GOTERM_MF_DIRECT	GO:0016836~hydro-lyase activity	4	4.545455	1.19E-04
GOTERM_MF_DIRECT	GO:0005518~collagen binding	5	5.681818	2.74E-04
GOTERM_MF_DIRECT	GO:0019901~protein kinase binding	10	11.36364	5.75E-04

GOTERM_MF_DIRECT	GO:0008270~zinc ion binding	13	14.77273	6.54E-0[illegible]
GOTERM_MF_DIRECT	GO:0004190~aspartic-type endopeptidase activity	4	4.545455	8.89E-0[illegible]
GOTERM_MF_DIRECT	GO:0050661~NADP binding	4	4.545455	9.54E-0[illegible]
GOTERM_MF_DIRECT	GO:0008083~growth factor activity	6	6.818182	0.001[illegible]
GOTERM_MF_DIRECT	GO:0034713~type I transforming growth factor beta receptor binding	3	3.409091	0.001[illegible]
GOTERM_MF_DIRECT	GO:0019904~protein domain specific binding	7	7.954545	0.001[illegible]
GOTERM_MF_DIRECT	GO:0046332~SMAD binding	4	4.545455	0.001[illegible]
GOTERM_MF_DIRECT	GO:0002020~protease binding	5	5.681818	0.001[illegible]
GOTERM_MF_DIRECT	GO:0043394~proteoglycan binding	3	3.409091	0.001[illegible]
GOTERM_MF_DIRECT	GO:0005125~cytokine activity	6	6.818182	0.001[illegible]
GOTERM_MF_DIRECT	GO:0048185~activin binding	3	3.409091	0.002[illegible]

GOTERM_MF_DIRECT	GO:0004222~metalloendopeptidase activity	5	5.681818	0.002[illegible]
GOTERM_MF_DIRECT	GO:0005102~receptor binding	8	9.090909	0.002[illegible]
GOTERM_MF_DIRECT	GO:0005516~calmodulin binding	6	6.818182	0.002[illegible]
GOTERM_MF_DIRECT	GO:0042277~peptide binding	4	4.545455	0.003[illegible]
GOTERM_MF_DIRECT	GO:0016301~kinase activity	6	6.818182	0.003[illegible]
GOTERM_MF_DIRECT	GO:0020037~heme binding	5	5.681818	0.005[illegible]
GOTERM_MF_DIRECT	GO:0005158~insulin receptor binding	3	3.409091	0.005[illegible]
GOTERM_MF_DIRECT	GO:0031490~chromatin DNA binding	4	4.545455	0.006[illegible]
GOTERM_MF_DIRECT	GO:0051117~ATPase binding	4	4.545455	0.009[illegible]
GOTERM_MF_DIRECT	GO:0019903~protein phosphatase binding	4	4.545455	0.009[illegible]
GOTERM_MF_DIRECT	GO:0018820~cyanamide hydratase activity	2	2.272727	0.009[illegible]

GOTERM_MF_DIRECT	GO:0051721~protein phosphatase 2A binding	3	3.409091	0.011[illegible]2[illegible]
GOTERM_MF_DIRECT	GO:0008234~cysteine-type peptidase activity	3	3.409091	0.013[illegible]6[illegible]
GOTERM_MF_DIRECT	GO:0030284~estrogen receptor activity	2	2.272727	0.013[illegible]6[illegible]
GOTERM_MF_DIRECT	GO:0051087~chaperone binding	4	4.545455	0.014[illegible]3[illegible]
GOTERM_MF_DIRECT	GO:0004713~protein tyrosine kinase activity	4	4.545455	0.015[illegible]0[illegible]
GOTERM_MF_DIRECT	GO:0008022~protein C-terminus binding	5	5.681818	0.015[illegible]8[illegible]
GOTERM_MF_DIRECT	GO:0008134~transcription factor binding	5	5.681818	0.015[illegible]3[illegible]
GOTERM_MF_DIRECT	GO:0004714~transmembrane receptor protein tyrosine kinase activity	4	4.545455	0.016[illegible]
GOTERM_MF_DIRECT	GO:0042803~protein homodimerization activity	9	10.22727	0.017[illegible]8[illegible]
GOTERM_MF_DIRECT	GO:0001223~transcription coactivator binding	3	3.409091	0.017[illegible]3[illegible]
GOTERM_MF_DIRECT	GO:0051879~Hsp90 protein binding	3	3.409091	0.017[illegible]3[illegible]

GOTERM_MF_DIRECT	GO:0043565~sequence-specific DNA binding	6	6.8181 82	0.0178 57
GOTERM_MF_DIRECT	GO:0030331~estrogen receptor binding	3	3.4090 91	0.0182 98
GOTERM_MF_DIRECT	GO:0048156~tau protein binding	3	3.4090 91	0.0182 98
GOTERM_MF_DIRECT	GO:0043539~protein serine/threonine kinase activator activity	3	3.4090 91	0.0182 98
GOTERM_MF_DIRECT	GO:0034714~type III transforming growth factor beta receptor binding	2	2.2727 27	0.0183 17
GOTERM_MF_DIRECT	GO:0004879~RNA polymerase II transcription factor activity, ligand-activated sequence-specific DNA binding	3	3.4090 91	0.0257 54
GOTERM_MF_DIRECT	GO:0004064~arylesterase activity	2	2.2727 27	0.0273 51
GOTERM_MF_DIRECT	GO:0051434~BH3 domain binding	2	2.2727 27	0.0273 51
GOTERM_MF_DIRECT	GO:0071253~connexin binding	2	2.2727 27	0.0318 37
GOTERM_MF_DIRECT	GO:0050660~flavin adenine dinucleotide binding	3	3.4090 91	0.0393 4
GOTERM_MF_DIRECT	GO:0097110~scaffold protein binding	3	3.4090	0.0403

ECT			91	9[illegible]
GOTERM_MF_DIRECT	GO:0097200~cysteine-type endopeptidase activity involved in execution phase of apoptosis	2	2.272727	0.040[illegible] 48[illegible]
GOTERM_MF_DIRECT	GO:0005024~transforming growth factor beta-activated receptor activity	2	2.272727	0.040[illegible] 48[illegible]
GOTERM_MF_DIRECT	GO:0009055~electron carrier activity	3	3.409091	0.041[illegible] 6[illegible]
GOTERM_MF_DIRECT	GO:0044877~macromolecular complex binding	6	6.818182	0.042[illegible] 1[illegible]
GOTERM_MF_DIRECT	GO:0003700~transcription factor activity, sequence-specific DNA binding	7	7.954545	0.043[illegible] 3[illegible]
GOTERM_MF_DIRECT	GO:0008237~metallopeptidase activity	3	3.409091	0.043[illegible] 2[illegible]
GOTERM_MF_DIRECT	GO:0002039~p53 binding	3	3.409091	0.043[illegible] 2[illegible]
GOTERM_MF_DIRECT	GO:0097199~cysteine-type endopeptidase activity involved in apoptotic signaling pathway	2	2.272727	0.045[illegible] 7[illegible]
GOTERM_MF_DIRECT	GO:0004675~transmembrane receptor protein serine/threonine kinase activity	2	2.272727	0.045[illegible] 7[illegible]
GOTERM_MF_DIRECT	GO:0097153~cysteine-type endopeptidase activity involved in apoptotic process	2	2.272727	0.045[illegible] 7[illegible]
GOTERM_MF_DIRECT	GO:0005114~type II transforming growth factor beta	2	2.2727	0.045[illegible]

ECT	receptor binding		27	73
GOTERM_MF_DIRECT	GO:0008201~heparin binding	4	4.5454<br>55	0.0478<br>71
GOTERM_MF_DIRECT	GO:0016004~phospholipase activator activity	2	2.2727<br>27	0.0495<br>78
GOTERM_MF_DIRECT	GO:0016175~superoxide-generating NADPH oxidase activity	2	2.2727<br>27	0.0539<br>62
GOTERM_MF_DIRECT	GO:0042301~phosphate ion binding	2	2.2727<br>27	0.0583<br>27
GOTERM_MF_DIRECT	GO:0045296~cadherin binding	5	5.6818<br>18	0.0583<br>66
GOTERM_MF_DIRECT	GO:0070411~I-SMAD binding	2	2.2727<br>27	0.0620<br>72
GOTERM_MF_DIRECT	GO:0008289~lipid binding	4	4.5454<br>55	0.0628<br>56
GOTERM_MF_DIRECT	GO:0003682~chromatin binding	6	6.8181<br>82	0.0651<br>51
GOTERM_MF_DIRECT	GO:0001228~transcriptional activator activity, RNA polymerase II transcription regulatory region sequence-specific binding	6	6.8181<br>82	0.0685<br>01
GOTERM_MF_DIRECT	GO:0051393~alpha-actinin binding	2	2.2727<br>27	0.0713<br>02

GOTERM_MF_DIRECT	GO:0005160~transforming growth factor beta receptor binding	2	2.272727	0.071302
GOTERM_MF_DIRECT	GO:0004707~MAP kinase activity	2	2.272727	0.071302
GOTERM_MF_DIRECT	GO:0004708~MAP kinase kinase activity	2	2.272727	0.079854
GOTERM_MF_DIRECT	GO:0046965~retinoid X receptor binding	2	2.272727	0.079854
GOTERM_MF_DIRECT	GO:0008656~cysteine-type endopeptidase activator activity involved in apoptotic process	2	2.272727	0.079854
GOTERM_MF_DIRECT	GO:0019900~kinase binding	3	3.409091	0.080546
GOTERM_MF_DIRECT	GO:0046982~protein heterodimerization activity	5	5.681818	0.092384
GOTERM_MF_DIRECT	GO:0016491~oxidoreductase activity	4	4.545455	0.098395
